# Biochar mitigates the peatland GHG dilemma under contrasting water table regimes: phase-dependent responses of CO_2_ and CH_4_ over a two-year study

**DOI:** 10.1007/s42773-026-00610-2

**Published:** 2026-04-21

**Authors:** Peduruhewa H. Jeewani, Jennifer M. Rhymes, Chris D. Evans, Davey L. Jones, David R. Chadwick

**Affiliations:** 1 School of Environmental and Natural Sciences, Bangor University, Bangor, Gwynedd LL57 2UW UK; 2 UK Centre for Ecology and Hydrology, Bangor, Gwynedd LL57 2UW UK

**Keywords:** Climate change, Greenhouse gas removal, Biochar, Nature-based solutions, Water table level

## Abstract

Peat soils are major terrestrial carbon stores, yet drainage alters redox conditions that stimulate CO_2_, CH_4_, and N_2_O emissions. We conducted a two-year mesocosm experiment to quantify how water table level (0, 20, and 40 cm) interacts with organic amendments to regulate gaseous C and N fluxes from lowland peat. Amendments included *Miscanthus* biochar, *Miscanthus* chip, paper waste, biosolids, and cereal straw. Results revealed that moderate drainage (WTL_20_) provided the optimal balance between carbon loss and CH_4_ suppression compared to saturated (WTL_0_). Although CO_2_ emissions increased under WTL_20_, CH_4_ fluxes declined by over 90% relative to WTL_0_, where methanogenesis dominated. This shift in the Control (from WTL_0_ toWTL_20_), reduced overall CO_2_-equivalent emissions by 17 t CO_2_eq ha^−1^ yr^−1^, highlighting the critical GHG balance of maintaining a WTL_20_ in lowland peatlands. Among the amendments, labile, low C:N amendments (cereal straw, biosolids) increased CO_2_ and N_2_O emissions under WTL_20_, indicating enhanced aerobic mineralization following oxygen exposure. In contrast, biochar consistently outperformed all treatments, reducing cumulative CO_2_ emissions by up to 52% compared with the Control-WTL_40_ when assessed over the full 730-day experimental period. The consistent reduction in GHG emissions indicates constrained peat carbon mineralization under biochar amendment across contrasting hydrological conditions (WTL_0_ and WTL_20_) in two consecutive years. Overall, this study demonstrates that integrating WTL_0_ and WTL_20_ water table manipulations alternatively with stable, recalcitrant amendments such as biochar substantially altered greenhouse gas fluxes, offering a promising strategy to mitigate emissions while adding to and maintaining peat carbon stocks and fluxes from lowland bare peat.

**Graphical Abstract:**

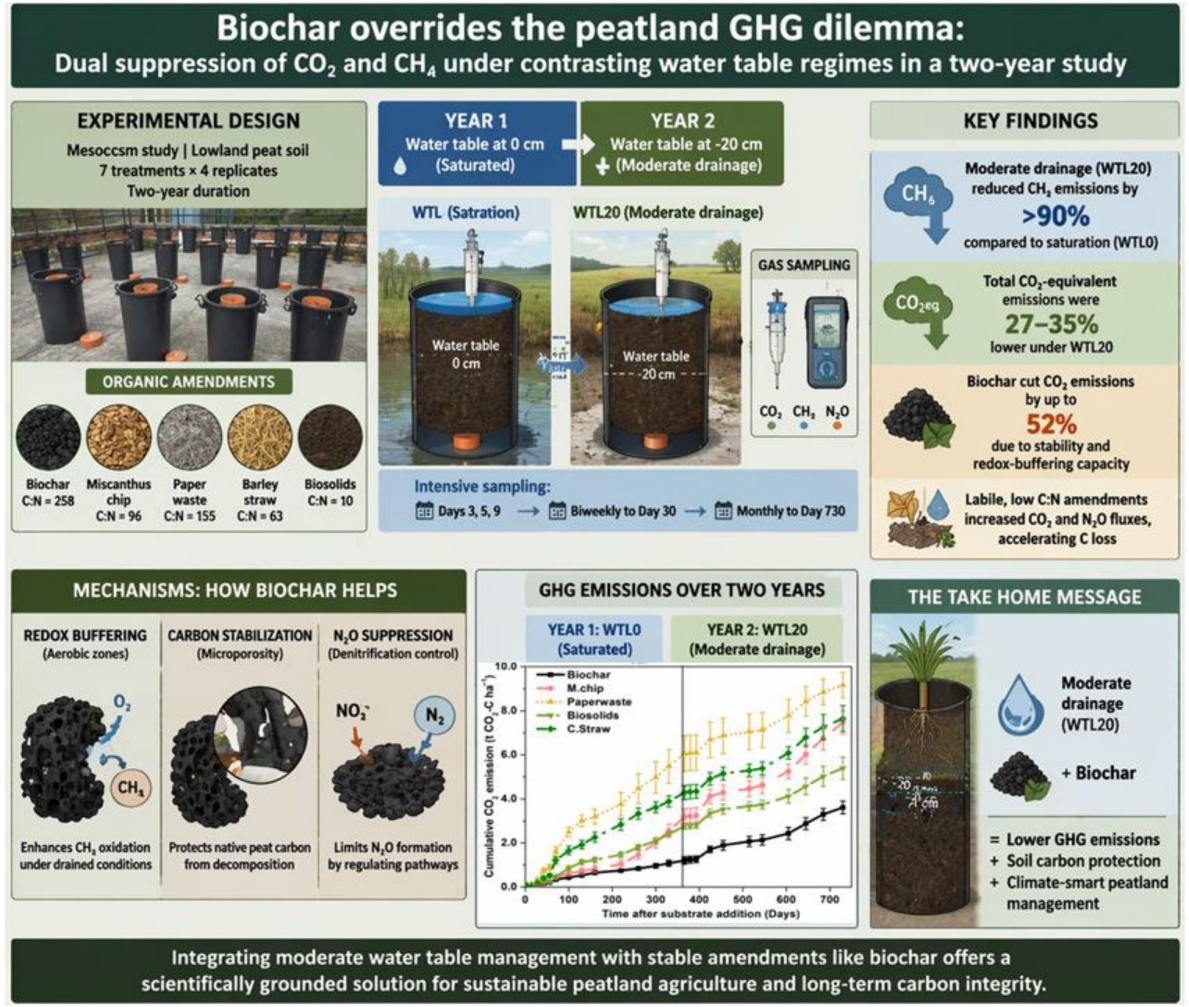

**Supplementary Information:**

The online version contains supplementary material available at 10.1007/s42773-026-00610-2.

## Highlights

- Moderate drainage (WTL_20_) reduced CH_4_ emissions by > 90% compared to saturation (WTL_0_).
- Total CO_2_eq emissions were 27–35% lower under WTL_20_, optimizing climate outcomes.
- Biochar cut CO_2_ emissions by up to 52% due to its stability and redox-buffering capacity.
- Labile, low C:N amendments increased CO_2_ and N_2_O fluxes, accelerating C loss.

**Supplementary Information:**

The online version contains supplementary material available at 10.1007/s42773-026-00610-2.

## Introduction

Following the goals established under the Paris Agreement to keep global temperature rise within 1.5–2 °C above pre-industrial levels, deep reductions in greenhouse gas (GHG) emissions are needed to reach net-zero emissions by 2050 (Zhai et al. 2018). Peatlands act as both a source and sink for GHGs, and thus have the potential to become an element of climate change mitigation (Leifeld and Menichetti 2018), with the potential to become net sinks for GHGs if optimally managed (Richard et al. 2021). Although peatlands occupy only about 0.3% of Earth’s land area, they account for an estimated 2–5% of human-induced GHGs (Leifeld and Menichetti 2018; Evans et al. 2021b). This highlights a critical need to determine and maintain optimal water table levels and best management practices in agricultural peatlands to minimise CO_2_ emissions whilst also avoiding elevated emissions of methane (CH_4_) and nitrous oxide (N_2_O).

Water table management in peatlands is widely considered to be the most important measure to curb peatland CO_2_ emissions (Günther et al. 2020; Evans et al. 2021a). Meta-analyses suggest that raising water tables by 10 cm reduces CO_2_ emissions by around 2.7 t CO_2_-C ha^−1^ yr^−1^ in tropical peatlands (Novita et al. 2021), while for field studies in Finland the equivalent reduction was 1.7 t CO_2_-C ha^−1^ yr^−1^ per 10 cm (Pearson et al. 2015), and for an analysis of eddy covariance data for UK peatlands it was 1.3 t CO_2_-C ha^−1^ yr^−1^ per 10 cm water table raise (Evans et al. 2021a, b). Raising water levels in peatlands can substantially reduce CO_2_ emissions by suppressing aerobic peat oxidation; however, this benefit may be partly offset by increased CH_4_ emissions (Evans et al. 2021a; Hu et al. 2024). When soils become saturated, restricted diffusion of atmospheric oxygen shifts microbial metabolism toward anaerobic pathways, promoting methanogenesis while limiting aerobic CH_4_ oxidation (Günther et al. 2020; Knox et al. 2021). Although CH_4_ emissions are an intrinsic component of natural wetland carbon cycling that predate anthropogenic climate change (Evans et al. 2021b; Petro et al. 2023), full rewetting of nutrient-enriched former agricultural peatlands can generate CH_4_ fluxes exceeding natural background levels. In such systems, prolonged surface inundation has been shown to produce CH_4_ emissions sufficiently large to offset, or in extreme cases outweigh, the climatic benefits of reduced CO_2_ emissions when expressed as CO_2_ equivalents using 100-year Global Warming Potentials (Evans et al. 2021a; Allan et al. 2023). Conversely, CH_4_ emissions decrease non-linearly to near-zero values when water tables are below 20–30 cm (Yang et al. 2013). To attain peatlands that are net climate-cooling over shorter time periods, and therefore achieve net GGR, it is therefore necessary either to augment rates of net CO_2_ uptake, or to suppress CH_4_ emissions below natural levels.

The quality and stoichiometry of organic substrates are key regulators of microbial activity and GHG production in peatlands (Manzoni et al. 2012; Evans et al. 2021a). Amendments with low C:N ratios and high substrate lability typically enhance microbial decomposition and nitrogen mineralization, stimulating CO_2_ and N_2_O emissions under oxic or intermittently oxic conditions, while also providing readily available carbon that can fuel methanogenesis when anaerobic microsites develop (Bridgham et al. 2013; Knox et al. 2021). In contrast, high C: N or chemically recalcitrant materials restrict microbial access to carbon, promote carbon stabilization through physical and chemical protection mechanisms, and reduce respiratory carbon losses (Lehmann et al. 2011; Jeewani et al. 2025a). In rewetted or partially drained peatlands, these substrate-driven effects interact strongly with water table position by regulating oxygen availability, redox dynamics, and dominant microbial pathways (Evans et al. 2021b; Allan et al. 2023). Accordingly, the present study employed a gradient of organic amendments spanning labile materials such as paper waste and cereal straw, intermediate substrates such as *Miscanthus* chips, to more processed and recalcitrant inputs including biosolids and biochar, in order to test how substrate quality modulates GHG responses under managed water table drawdown. While these and other studies show promise, particularly in relation to biochar application, all studies undertaken to date have been short-term, running for a year at most, and often less. As a result, the longer-term stability of applied organic matter and its sustained impacts on CO_2_, CH_4_ and N_2_O fluxes from re-wetted peat are largely unknown.

This study builds upon the one-year investigation by Jeewani et al. (2025a, b), which examined GHG fluxes and carbon balance under raised water-table and biochar-amended peatland conditions. The lowering of the peatland water table can trigger non-linear transitions in biogeochemical functioning when surface peat becomes hydraulically disconnected from the saturated zone. Once the upper peat layer desiccates, anaerobic porewater-mediated processes stop, and carbon cycling becomes dominated by aerobic mineralization. Such threshold-driven transitions represent a distinct biogeochemical phase rather than a continuation of pre-drawdown conditions. In the present study, Year 2 was characterized by complete drying of the upper 20 cm of peat, marking a shift to an oxidative surface-peat regime that is fundamentally different from the hydrologically connected conditions described in Jeewani et al. (2025a, b). We targeted a water table depth of approximately 20 cm below the soil surface, which is representative of management-relevant conditions in such systems and allows evaluation of the effects on CO_2_ and CH_4_ fluxes while maintaining cropping potential (Musarika et al. 2017). We tested three hypotheses: (1) that lowering the water table would shift GHG emissions toward CO_2_ dominance, in accordance with established peatland biogeochemical theory, and that the magnitude of this shift would be modulated by organic amendments; (2) that water table lowering to − 20 cm (WTL_20_) in Year 2 would reduce cumulative GHG-equivalent emissions relative to Year 1 (WTL_0_) in all treatments and; (3) among the amendments evaluated, biochar would result in the greatest mitigation of GHG emissions compared with unamended controls (WTL_20_ and WTL_40_) after 730 days under both water table regimes.

## Materials and methods

### Site description

Undisturbed peat soil mesocosms were obtained in May 2022 from an intensively managed agricultural field situated on lowland fen peat at the Lapwing Estate, Doncaster, UK (53° 27′ N, 0° 54′ W). The site comprises historically drained fen peatland characterized by a 40–80 cm organic horizon overlying mineral substrate, classified as an Ombric Sapric Histosol (WRB 2014). Drainage initiated in the seventeenth century has led to significant and ongoing peat oxidation and subsidence. Over the preceding two decades, the field underwent intensive rotational cropping, primarily featuring *Brassica* species, wheat as a break crop, and a period of grass cover. The area is characterized by a temperate climate, with an average yearly temperature of 10.3 °C and annual precipitation totaling 1162 mm. To preserve soil structure integrity, sharpened PVC cylinders (20 cm internal diameter, 60 cm height) were used. The intact peat cores were subsequently extracted using mechanical excavation, transported to Bangor University, and maintained outdoors under ambient conditions for the entire 730-day experimental duration.

### Experimental design

Following the earlier findings of Jeewani et al. (2025a, b) on GHG emissions from organically amended peat mesocosms under high water table conditions, the present study investigates how a subsequent 20 cm lowering of the water table alters these emission dynamics and overall carbon balance. The mesocosm study included seven treatments, each replicated four times, with five of these treatments consisting of organic amendments spanning a range of C:N ratios. Five organic amendments were applied at the start of the study: (a) *M. giganteus* biochar was pyrolyzed at 450 °C for 30 min in a muffle furnace to produce biochar (~ 2 cm particle size) with a C:N ratio of 258); (b) *Miscanthus giganteus*-derived chip (size ranging from 1 to 2 cm; C:N ratio = 96); (c) paper waste obtained from commercial paper manufacturing (Ahlstrom Chirnside Ltd., Manchester, UK; C:N = 155); (d) barley straw (*Hordeum vulgare* L.; C:N = 63); and (e) anaerobically digested biosolids from a major urban wastewater treatment facility (C:N = 10). Together, these amendments spanned a broad gradient of C:N ratios, ranging from highly labile material (low C:N, biosolids) to more recalcitrant substrates (high C:N, biochar), representing contrasting qualities of organic matter inputs (Ghosh and Leff 2013; Siedt et al. 2021; Marmier et al. 2022; Leopard et al. 2025). Two control treatments were included: (1) a dynamic control transitioning from a water table at the soil surface (WTL_0_ year 1) to one at 20 cm below the surface (WTL_20_, year 2) to simulate moderate drainage conditions, and (2) a static control maintained with a water table 40 cm below the soil surface (WTL_40_) for both years representing the ‘business-as-usual’ (BAU) management. Each mesocosm was positioned within a larger container equipped with drainage holes to keep the water table at − 20 cm (Additional file 1: Fig. S1). All cores were maintained without vegetation. Water table depths (WTL_20_ and WTL_40_) were maintained using drainage holes installed at the corresponding depths to remove excess water, ensuring that the water table remained largely stable throughout the rainfall season. Natural rainfall sustained the water table, with supplemental tap water added during dry periods as needed. Water table levels were monitored regularly, and a schematic of the control system is provided in Additional file 1: Fig. S1. All organic materials were added at an application rate equivalent to 20 t C ha^−1^ (Jones et al. 2012; Pandit et al. 2018) and were mixed by hand into the top 10 cm of soil to simulate field-based application. No additional amendments were applied, and apart from water levels the experiment was managed as in Year 1. At the beginning of both Year 1 and Year 2, we conducted intensive sampling on days 3, 5, 9, then biweekly until day 30 at which point sampling continued monthly up to 12 months. A variable sampling intensity was chosen to ensure that the GHG fluxes from amendments (Year 1) and water table adjustment (Year 2) were adequately captured. The characteristics of the soil and organic amendments were published in (Jeewani et al. 2025a).

### Soil GHG flux measurements and calculations

A gas-tight PVC chamber (20 cm inner diameter × 4 cm height) with a Suba-Seal^®^ septum (Sigma-Aldrich Ltd., UK) was placed on each mesocosm during sampling, enclosing a headspace of 3145 cm^3^ (Additional file 1: Fig. S1). Gas samples (20 mL) from the headspace were taken at 0, 20, and 40 min after sealing, using airtight polypropylene syringes and immediately injected into pre-evacuated 20 mL Exetainer^®^ vials (Fisher Scientific, Denmark). The levels of CO_2_, CH_4_, and N_2_O were measured via gas chromatography (Greenhouse gas autosampler AS-210, SRI Instruments Europe, Germany). Fluxes were derived from the linear change in gas concentration over the 40-min period, corrected for the chamber volume-to-surface-area ratio and ambient temperature, following the approach outlined by Sánchez-Rodríguez et al. (2019).

1 $$F=\frac{\Delta c}{\Delta t} \times AV\times R\times TP$$

where *F* = Gas flux (e.g., µmol m^−2^ s^−1^ or mg m^−2^ h^−1^), Δ*c*/Δ*t* = Rate of change in gas concentration over time (slope of concentration vs. time), *V* = Volume of the chamber (m^3^), *A* = Surface area covered by the chamber (m^2^), *P* = Atmospheric pressure (Pa), *R* = Universal gas constant (8.314 J mol^−1^ K^−1^), *T* = Temperature in Kelvin (K).

GHG emissions were calculated by subtracting the gas concentrations at time 0 from those measured 60 min later, with adjustments made for temperature and the ratio of chamber volume to soil surface area. Cumulative emissions of CO_2_, N_2_O, and CH_4_ were calculated by linear interpolation of measured flux rates (Wen et al. 2019).

2 $$\text{Cumulative\; emissions }= \sum \_i^n[\left(\frac{{R}_{i-1}+{R}_{i}}{2}\times Di\right)]$$

where *R*_*i*_^−1^ and *R*_*i*_ represent the GHG flux rates at the (i − 1)th and ith sampling, *D*_*i*_ is the number of days between these two samplings, and *n* is the total number of sampling events.

To calculate the total GHG emissions and enable comparison across treatments, the GHGs were expressed as CO_2_ equivalents (CO_2_eq) using GWP conversion factors: 265 for N_2_O and 28 for CH_4_, according to IPCC (2023).

3 $${\mathrm{Total}}\;{\mathrm{GHG}}\;{\mathrm{emissions}}\;\left( {{\mathrm{CO}}_{2} \;{\mathrm{equivalent}}} \right) = {\mathrm{CO}}_{2} + \left( {265 \times {\mathrm{N}}_{2} {\mathrm{O}}} \right) + \left( {28 \times {\mathrm{CH}}_{4} } \right)$$

The carbon balance of each mesocosm was determined using the following equation:

4 $$\text{Original\; C\; content}={\sum }_{\mathrm{i}=1}^{4}{C}_{\mathrm{i}}\times {P}_{\mathrm{i}}$$

where *C*_*i*_ denotes the carbon content of each treatment (t C ha^−1^), and *P*_*i*_ represents the fraction of the total soil mass corresponding to that treatment.

5 $$\text{Total\; C\; content}=\text{Native\; soil\; C\; content}+\text{C\; addition}$$

6 $$\text{C\; loss}=\text{Cumulative\; emissions\; of }\;{\mathrm{CO}}_{2}+\text{Cumulative\; emissions \; of }\;{\mathrm{CH}}_{4}$$

Assuming no DOC losses (cores were not flushed during the experiment), carbon losses from the mesocosms were estimated by adding the total CO_2_ and CH_4_ fluxes. Carbon storage was determined as the difference between the initial carbon content and the total carbon loss, using the following formula:

7 $$\text{C\; storage}=\text{Total\; C\; content}-\text{C\; loss}$$

### Statistical analysis

Greenhouse gas data collected over two years were analyzed using repeated measures ANOVA in R. Data normality and homogeneity of variances were assessed using the Shapiro–Wilk and Levene’s tests, respectively. When assumptions were violated, CO_2_ and CH_4_ flux data were log-transformed to meet normality and variance homogeneity. Other variables met assumptions without transformation. Differences among treatment means were assessed using a one-way ANOVA followed by Tukey’s HSD test at the 95% confidence level, conducted in SPSS v24 (SPSS Inc., Chicago, IL, USA). Relationships between site-specific soil properties and the annual cumulative GHG fluxes as well as the mitigation potential from rewetting were examined using multiple linear regression with Pearson correlation. Unless stated otherwise, results are presented as means with their standard errors (n = 4). Results were considered statistically significant at *p* < 0.05, and only these findings are discussed. Data were visualized using Origin 2022 (Origin Lab Corp., USA).

## Results

### Effect of water table depth and C amendment on greenhouse gas emissions

#### CO_2_ emission

**Fig. 1:**
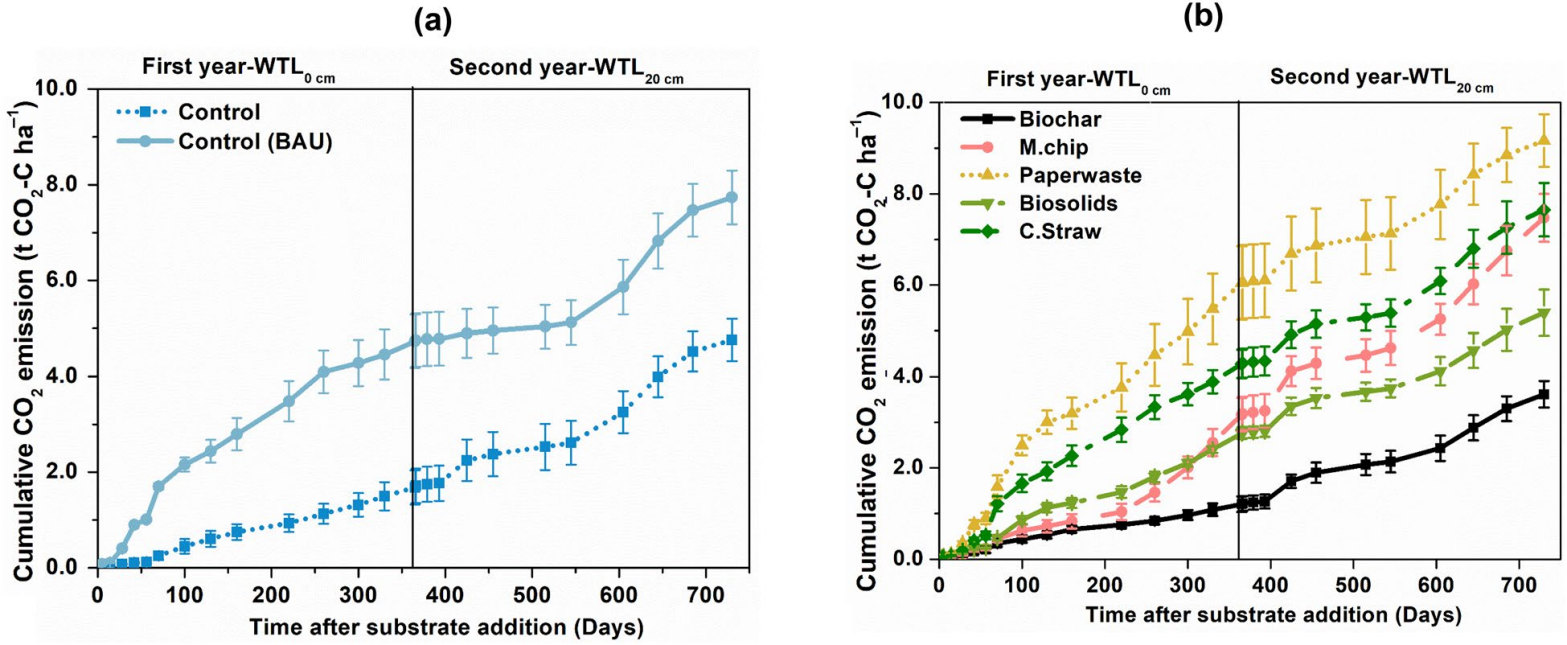
Temporal dynamics of CO_2_ fluxes under rewetted (Year 1) and drained (Year 2) conditions across soil amendments. Cumulative CO_2_ emissions from controls (**a**) and organic amendments (**b**) at both water table levels. The organic amendments included *Miscanthus* biochar (Biochar), *Miscanthus* chips (M.chip), paper waste, biosolids and cereal straw (C.Straw). The water table level (WTL) was at “the soil surface” (0 cm; WTL_0_, saturated) for year 1 and at 20 cm (WTL_20_, moderately drained) in year 2. The water table depth for the BAU Control was 40 cm (TL_40_) throughout the two-year experimental period. Values represent mean ± standard errors (*n* = 4)

The cumulative CO_2_ emission of the Control-WTL_40_, which had a continuous 40 cm water table level for two years, was 7.70 ± 0.56 t CO_2_-C ha^−1^ (Fig. 1a). For comparison, the Control-WTL_20_ treatment showed cumulative emissions of 4.76 ± 0.43 t CO_2_-C ha^−1^ at the end of the second year, representing a 39% decrease compared to Control-WTL_40_ (*p* < 0.05) (Fig. 1a). A pronounced increase in CO_2_ emissions was detected at the point of drainage, particularly in the Control WTL_20_ treatment, where emissions reached 6.5 g CO_2_ m^−2^ during days 356–375 (Fig. 1b and Additional file 1: Fig. S2). The biochar treatment with WTL_20_ exhibited lowest cumulative CO_2_ emissions among all treatments, releasing 3.61 ± 0.30 t CO_2_-C ha^−1^ at WTL_20_ at the end of year 2 (Fig. 1b). Cumulative CO_2_ emissions during the second year alone were 2.4 t CO_2_-C ha^−1^, representing a two-fold increase compared to WTL_0_ in year 1. Notably, paper waste, straw, and biosolids showed elevated cumulative CO_2_ emissions (9.1 ± 0.6, 7.6 ± 0.6, and 5.3 ± 0.5 t CO_2_-C ha^−1^, respectively), while biochar maintained near-baseline fluxes at 3.61 ± 0.3 t CO_2_-C ha^−1^ (Fig. 1b). During the WTL_20_ phase (days 366–730), biochar addition resulted in a 13% increase in cumulative CO_2_ emissions relative to the corresponding Control WTL_20_ (*p* = 0.042); however, when emissions were integrated over the entire 730-day experimental period, biochar consistently outperformed all treatments, reducing cumulative CO_2_ emissions by up to 52% compared with the Control WTL_20_ (Fig. 1).

#### CH_4_ emission

**Fig. 2:**
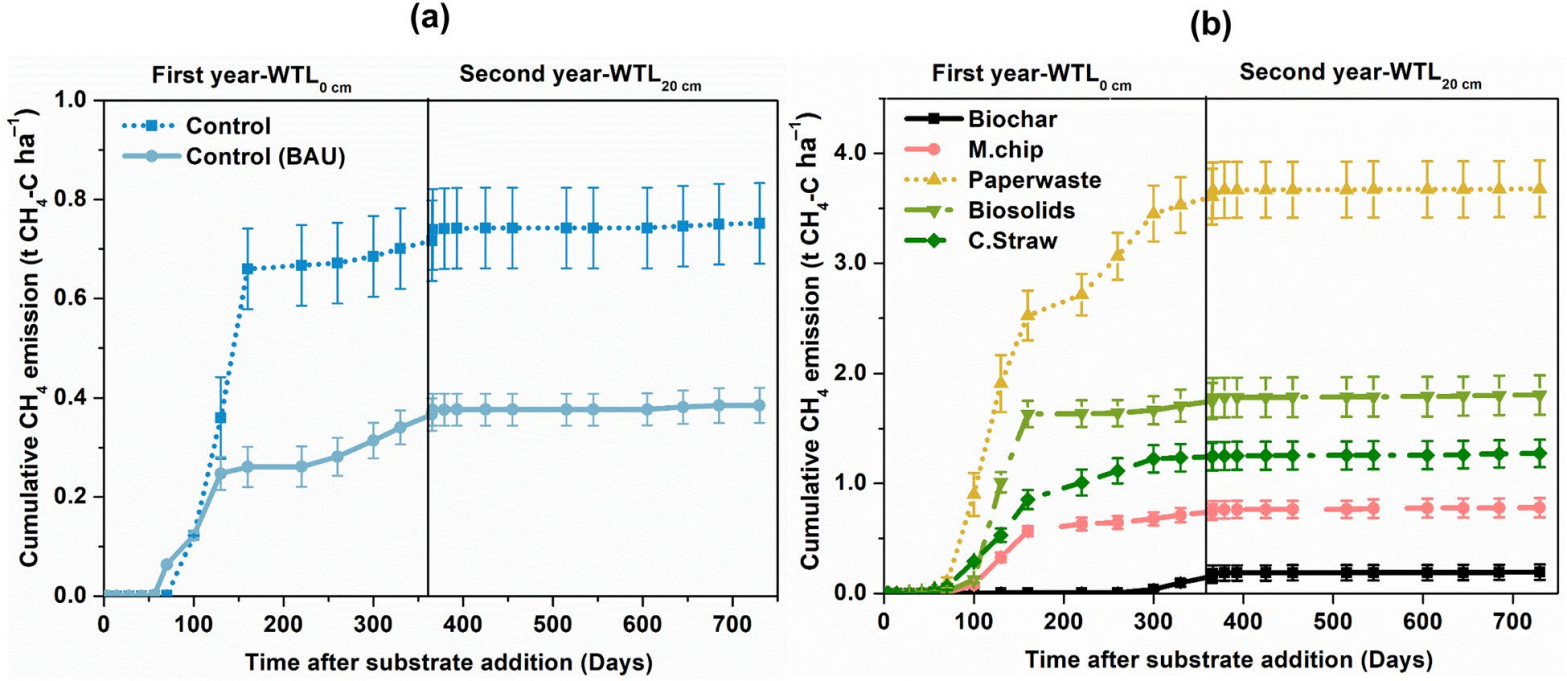
Temporal dynamics of CH_4_ fluxes under rewetted (Year 1) and drained (Year 2) conditions across soil amendments. Cumulative CH_4_ emissions from controls (**a**) and organic amendments (**b**) at both water table levels. The organic amendments included *Miscanthus* biochar (Biochar), *Miscanthus* chips (M.chip), paper waste, biosolids and cereal straw (C.Straw). The water table level was at te soil surface (0 cm; WTL_0_, saturated) for year 1 and at 20 cm (WTL_20,_ moderate drainage) in year 2. The water table depth for the BAU Control was 40 cm (WTL_40_) throughout the two-year experimental period. Values represent mean ± standard errors (*n* = 4). Note the different y-axis scales for panel **a** and panel **b**

In the second year, the Control-WTL_40_ and WTL_20_ treatments exhibited substantial reductions in cumulative CH_4_ emissions, reaching approximately 0.004 t CH_4_-C ha^−1^ (Fig. 2a). While amendments notably influenced emission dynamics at WTL_0_ in the first year, draining the water table to 20 cm in the second year effectively suppressed CH_4_ emissions across all treatments to negligible levels (< 0.02 mg CH_4_ m^−2^ d^−1^). Quantitatively, maintaining the water table at 20 cm resulted in a 98% reduction in CH_4_ emissions relative to the WTL_0_.

#### N_2_O emission

**Fig. 3:**
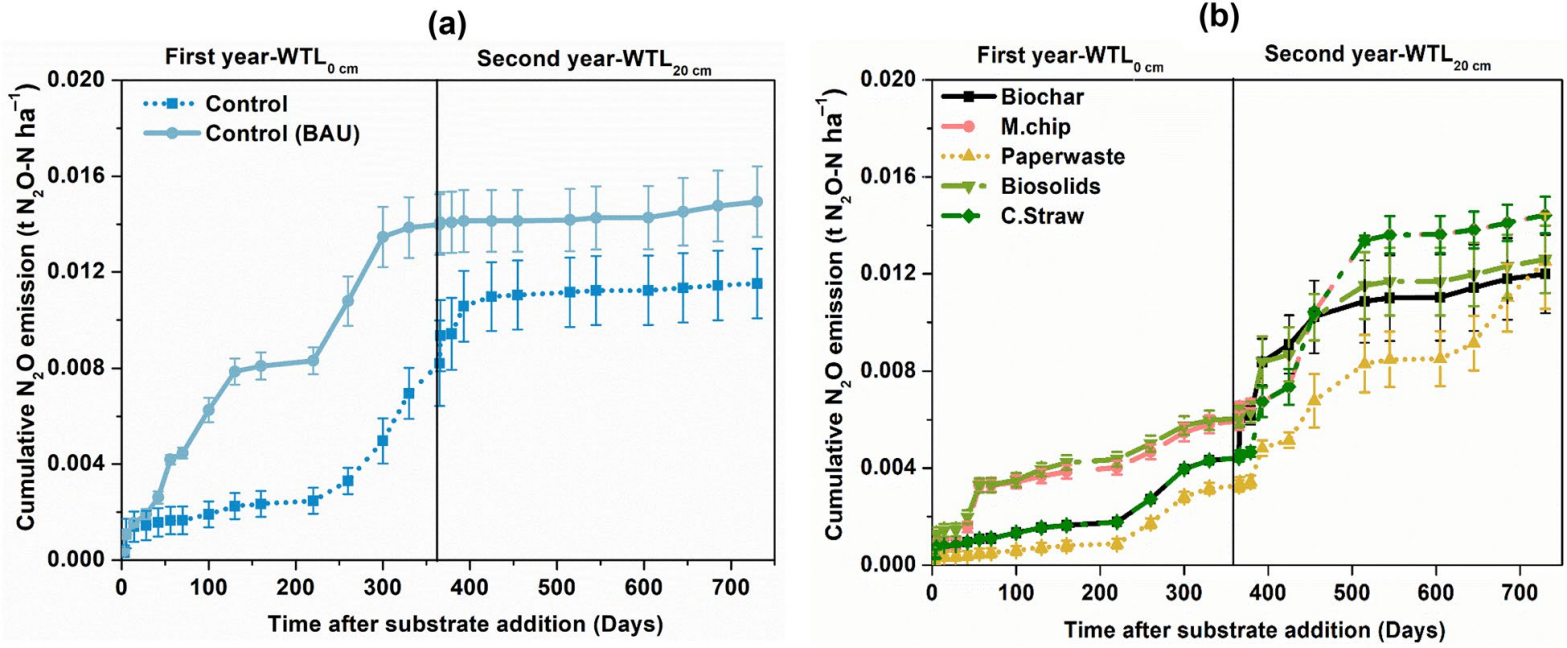
Temporal dynamics of N_2_O fluxes under rewetted (Year 1) and drained (Year 2) conditions across soil amendments. Cumulative N_2_O emissions from controls (**a**) and organic amendments (**b**) at both water table levels. The organic amendments included *Miscanthus* biochar (Biochar), *Miscanthus* chips (M.chip), paper waste, biosolids and cereal straw (C.Straw). The water table level was at te soil surface (0 cm; WTL_0_, saturated) for year 1 and at 20 cm (WTL_20_, moderately drained) in year 2. The water table depth for the BAU Control was 40 cm (WTL_40_) throughout the two-year experimental period. Values represent mean ± standard errors (*n* = 4)

Figure 3b summarises the cumulative N_2_O emissions over the two-year period. In the second year, N_2_O emissions from the control treatments (WTL_20_ and WTL_40_) remained at negligible levels (approximately 0.003 t N_2_O-N ha^−1^).

However, when the water table shifted from 0 to 20 cm depth, organic amendment treatments resulted in marked increases in N_2_O emission dynamics (Fig. 3b). For example, N_2_O emission from the cereal straw treatment increased gradually from 0.002 to 0.016 t N_2_O-N ha^−1^. Relative to the Control-WTL_20_ treatment, in the second year the biochar, *Miscanthus* chip, paper waste and cereal straw treatments increased N_2_O cumulative emissions by 53%, 60%, 67%, and 71%, respectively. The biochar treatment showed a steady but less pronounced increase, reaching cumulative emissions of approximately 0.012 t N_2_O-N ha^−1^ by the end of the experiment.

### Net GHG emissions and C balance

**Fig. 4:**
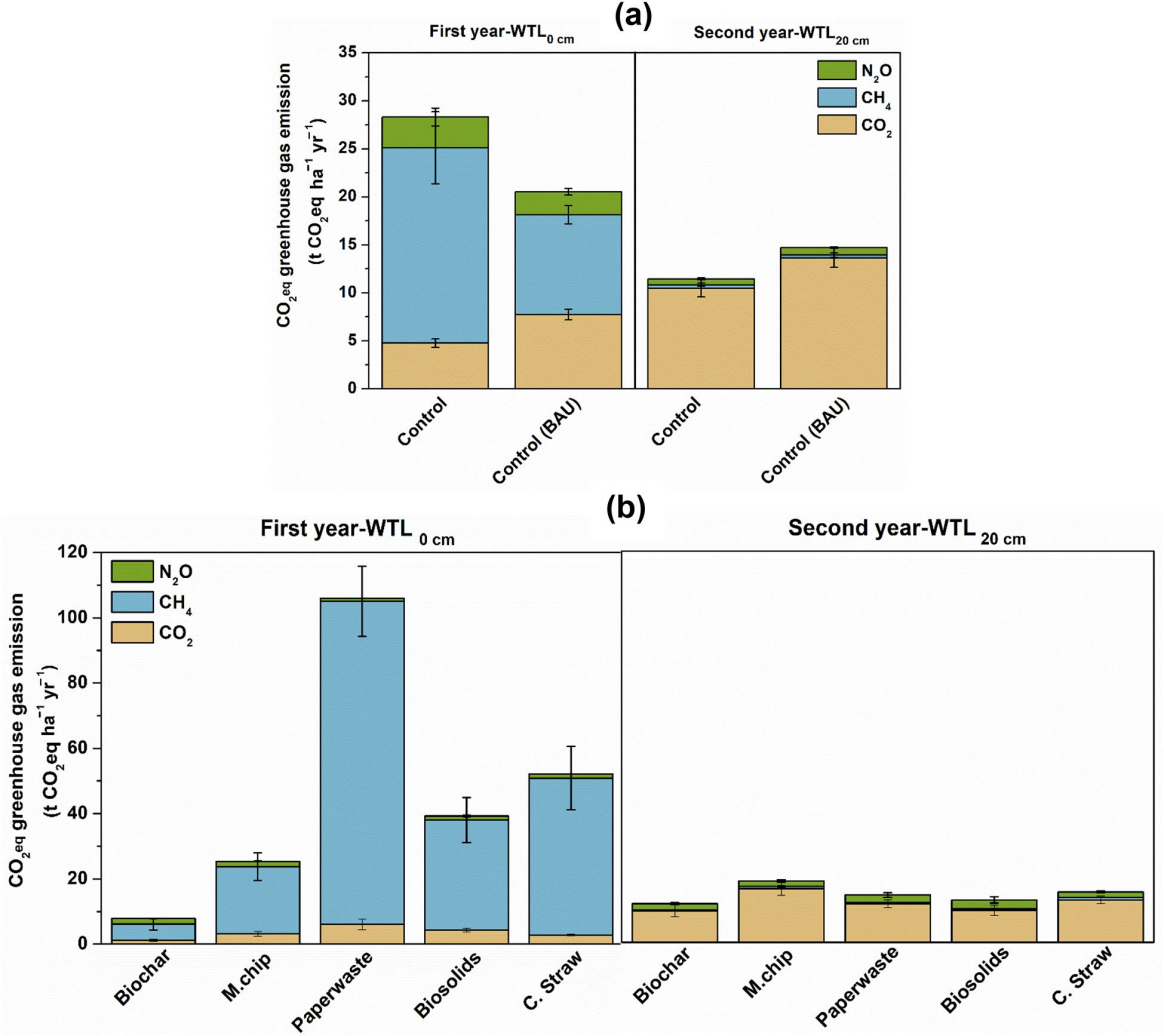
Effect of organic carbon amendment on greenhouse gas emissions when expressed in CO_2_ equivalents (a and b). GWP was based on radiative forcing over a 100-years’ time horizon: CO_2_ = 1, CH_4_ = 28, and N_2_O = 265. The carbon amendments included *Miscanthus* biochar (Biochar), *Miscanthus* chips (M.chip), paper waste, biosolids and cereal straw (C.Straw). The water table level was at the soil surface (0 cm; WTL_0_, saturated) for year 1 and at 20 cm (WTL_20_, moderately drained) in year 2. The water table depth for the BAU Control was 40 cm (WTL_40_) throughout the two-year experimental period. Values represent mean ± standard errors (*n* = 4). Note the different y-axis scales for panel **a** and panel **b**

When expressed as CO_2_ equivalents (CO_2_eq) over 100-year time horizons, annual cumulative GHG emissions varied considerably among the controls, ranging from 20.5 to 28.3 t CO_2_eq ha^−1^ yr^−1^ in WTL_0_ within year 1 to 11.7–13.7 in the WTL_20_ treatment in the second year (Fig. 4a and Table 1). In the WTL_0_ control, CH_4_ contributed > 70% of total CO_2_eq GHG emissions across all treatments, whereas CO_2_ dominated emissions in the second year in the Control WTL_20._ The cumulative CO_2_ emissions of biochar treatment were 9.67 t CO_2_eq ha^−1^ yr^−1^, resulting in an overall net GHG emission of 11.9 t CO_2_eq ha^−1^ yr^−1^ at WTL_20_.

**Table 1:** Carbon and greenhouse gas (GHG) balance with respect to organic C amendment in an agricultural peat soil after two years

Treatment	Biomass C added (t C ha^−1^)	Biomass C added (t CO_2_ ha^−1^)	Cumulative CO_2_ flux (t CO_2_e ha^−1^)	Cumulative CH_4_ flux (t CO_2_e ha^−1^)	Cumulative N_2_O flux (t CO_2_e ha^−1^)	C balance (t)	GHG balance (t CO_2_e ha^−1^)	Net CO_2_ difference vs control-(BAU) (t CO_2_e ha^−1^)	Net GHG difference versus control (t CO_2_e ha^−1^)
Biochar	20	73.3	13.3	7.3	5.6	− 16.19	− 47.2	− 23.7	− 94.2
M. chip	20	73.3	27.4	29.2	5.1	− 11.74	− 11.6	− 21.2	− 65.5
C. Straw	20	73.3	28.1	47.6	4.8	− 11.07	7.1	− 19.6	− 43.7
Biosolids	20	73.3	19.8	67.4	6.1	− 12.8	20	− 20.6	− 29.6
Paper waste	20	73.3	33.6	137.5	5.3	− 7.15	103.1	− 15.4	52.4
Control	0	0.0	17.5	28.1	4.9	5.51	50.5	− 2.7	2.9
Control (BAU)	0	0.0	28.4	14.4	3.6	8.12	46.4	0.0	0.0

The C amendments included *Miscanthus* biochar, *Miscanthus* chips (M.chip), paper waste, biosolids, and cereal straw (C.Straw). The experiment had two controls without C amendments; a business-as-usual (BAU) control treatment with a water table level of 40 cm below the soil surface (WTL_40_) throughout the two-year experimental period, and a second control treatment with water table of 0 cm (WTL_0_, saturated) in year 1 that transitioned to a water table at 20 cm (WTL_20_, moderately drained) in year 2. All values presented are cumulative values after two years of experimental duration. Soil column depth was 50 cm and bulk density was 0.52 ± 0.05. Values represent mean ± standard errors (*n* = 4). Emissions of CH_4_ and N_2_O were converted to CO_2_ equivalents based on their respective 100-year global warming potentials (IPCC Assessment Report: Climate Change 2023)

By the end of the second year we calculated soil C loss assuming no DOC loss from the mesocosms. Soil C loss was lowest with biochar treatment (4.7 t C ha^−1^ yr^−1^) but markedly higher in the paper waste and cereal straw treatments (11.2 and 8.1 t C ha^−1^ yr^−1^, respectively) (*p* = 0.004) (Additional file 1: Fig. S3). In the Control treatments, total C storage after year 2 was 166.1 t C ha^−1^, indicating a net soil C loss of 6.3 t C ha^−1^ over the two-year duration (*p* = 0.021) (Additional file 1: Fig. S3).

### Water table depth on GHG emission

**Fig. 5:**
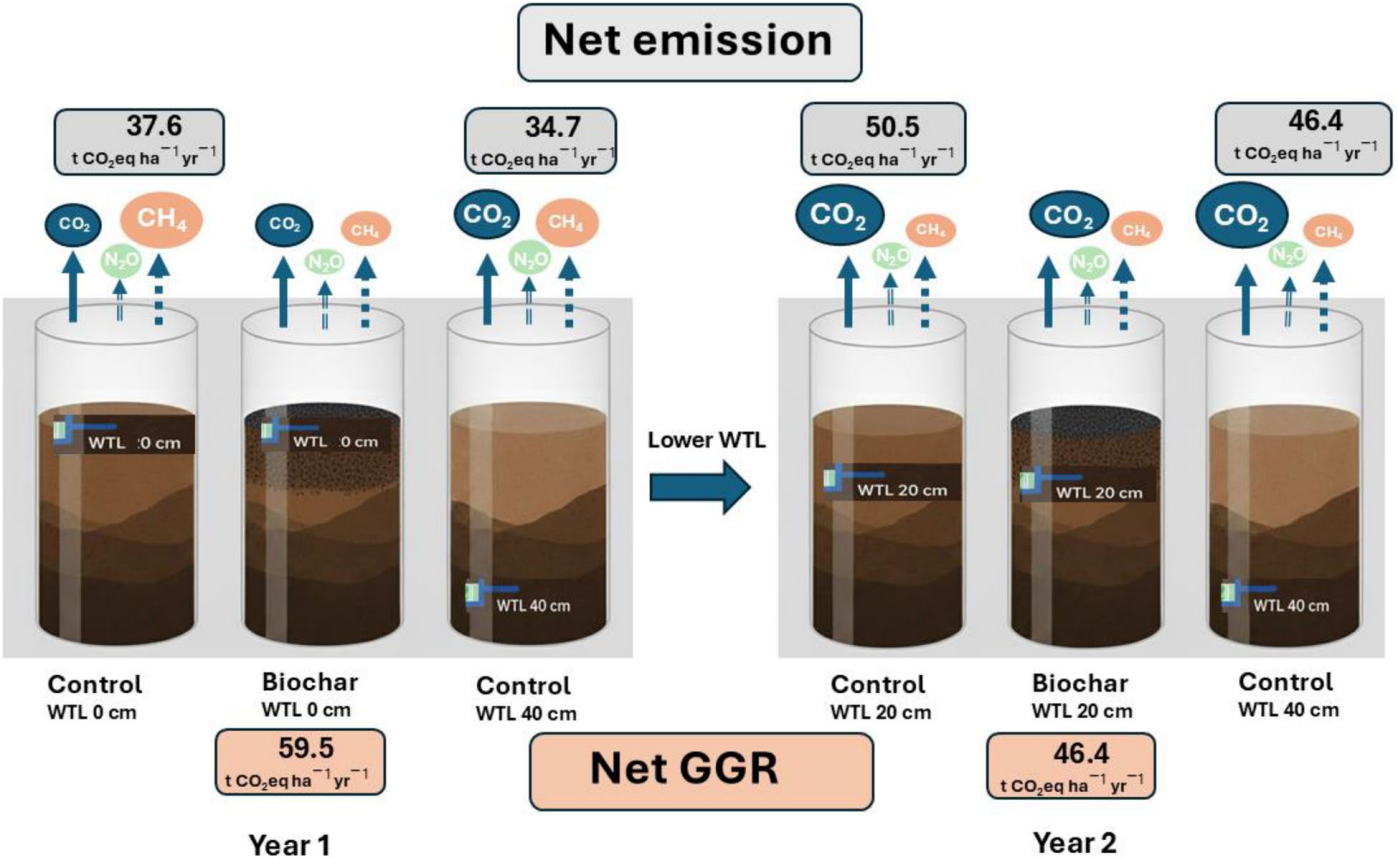
Effects of water table level (WTL) management and biochar amendment on net greenhouse gas (GHG) emissions from lowland peat soils over a two-year mesocosm experiment. Net emissions are expressed as CO_2_ equivalents (t CO_2_eq ha^−1^ yr^−1^), integrating the global warming potential of CO_2_, CH_4_, and N_2_O fluxes. In Year 1, three treatments were applied: Control (WTL 0 cm), Biochar (WTL 0 cm), and Control (WTL 40 cm). In Year 2, following WTL lowering to 20 cm in the first two treatments, emissions were measured under Control (WTL 20 cm), Biochar (WTL 20 cm), and Control (WTL 40 cm). Biochar amendment significantly reduced net GHG emissions relative to control treatments at equivalent WTLs. Net greenhouse gas balance decreased from 59.5 t CO_2_eq ha^−1^ yr^−1^ in Year 1 to 46.4 t CO_2_eq ha^−1^ yr^−1^ by the end of Year 2, corresponding to emission reductions of 37–42% compared to controls. Arrows indicate the relative magnitude of individual gas fluxes, with solid lines representing CO_2_, dashed lines representing N_2_O, and dot-dashed lines representing CH_4_

Results from the Control peat mesocosms (WTL_0_ in year 1, WTL_20_ in year 2, and WTL_40_ in both years) revealed clear relationships between water table depth and GHG emissions (Fig. 5). Cumulative CO_2_ emissions were lowest at WTL_0_, ranging between 1.7 and 2.3 t CO_2_-C ha^−1^. At WTL_20_, cumulative CO_2_ emissions increased significantly to approximately 2.5–3.6 t CO_2_-C ha^−1^. The highest cumulative CO_2_ emissions occurred at WTL_40_, ranging from 4 to 5.4 t CO_2_-C ha^−1^. Cumulative CH_4_ emissions showed the opposite trend, with the highest emissions ay WTL_0_ (approximately 0.6–1 t CH_4_-C ha^−1^). At WTL_20_, cumulative CH_4_ emissions decreased considerably to 0.3–0.4 t CH_4_-C ha^−1^. The lowest cumulative CH_4_ emissions were observed at WTL_40_, at almost negligible levels (close to 0.02 t CH_4_-C ha^−1^). Cumulative N_2_O emissions were lowest at WTL_0_ (0.003 t N_2_O-N ha^−1^). At WTL_20_, cumulative N_2_O emissions increase to approximately 0.004–0.006 t N_2_O-N ha^−1^. The highest cumulative N_2_O emissions were observed at WTL_40_ (0.012 t N_2_O-N ha^−1^).

Based on the Pearson correlation coefficients (*r*) between soil properties and cumulative GHG fluxes, DOC and CO_2_ showed a strong positive correlation (*r* = 0.02, *p* < 0.001), soil organic matter showed a strong negative correlation with mineral associated organic matter (*r* = − 0.63, *p* < 0.001) and a moderate negative correlation with EC (*r* = − 0.43, *p* = 0.023; Additional file 1: Table S1). Weak but marginal relationships were observed between DOC and CH_4_ emissions (*p* < 0.10).

## Discussion

This two-year mesocosm experiment provided a unique opportunity to assess how water table variations influence GHG emissions from peat soils. During the first year, the system was maintained at WTL_0_ (saturation), while in the second year, it transitioned to WTL_20_ (moderate drainage). The comparison between these two hydrological regimes revealed marked differences in peat soil behaviour, particularly in terms of CO_2_, CH_4_, and N_2_O fluxes.

### Hydrological legacy effect on CO_2_ emissions

Water table level exerts a dominant and lasting influence on CO_2_ emissions from peat soils, with flux patterns shaped by both current hydrological status and the legacy effects of prior management (Evans et al. 2021a, 2023; Yang et al. 2025). In this study, lowering the water table from WTL_0_ (saturation) to a moderately drained condition at WTL_20_ resulted in a marked increase in CO_2_ emissions, approximately 3.1 ± 0.1 t CO_2_-C ha^−1^ in the Control treatment. This substantial rise aligns with the activation of aerobic microbial metabolism under enhanced oxygen availability, as also reported by Mäkiranta et al. (2012) and Evans et al. (2021a, 2023). Laboratory experiments have demonstrated that peak CO_2_ release typically occurs under moderate drying (− 20 to − 60 cm soil water level), conditions that optimize microbial respiration without severely restricting substrate diffusion (Säurich et al. 2019; Günther et al. 2020). Similarly, meta-analyses show that a 10 cm decline in water table can increase CO_2_ emissions by about 2.7 t CO_2_-C ha^−1^ yr^−1^ (Novita et al. 2021), while Finnish field data indicate a 0.17 t CO_2_-C ha^−1^ yr^−1^ rise per centimetre of drawdown (Pearson et al. 2015), both consistent with the magnitude observed in the WTL_20_ treatment.

In contrast, the long-term Control maintained at WTL_40_ over two years exhibited elevated yet comparatively lower CO_2_ fluxes than WTL_20_, suggesting that temporal feedbacks such as microbial adaptation and substrate depletion may constrain continued respiration under prolonged drainage. Similar attenuation of CO_2_ release has been observed in long-term drainage studies, where extended aeration reduces labile carbon pools and alters microbial stoichiometry (Jiao et al. 2024; Sun et al. 2025). Thus, while WTL_20_ can initially stimulate CO_2_ emissions through enhanced aerobic decomposition, constant deep drainage may lead to diminishing carbon losses as substrates become depleted. These findings highlight that the relationship between water table and CO_2_ flux is nonlinear and temporally dynamic, highlighting the need for adaptive water table management to balance carbon outcomes and maintain peatland ecosystem integrity.

### Substrate decomposability hierarchy

The decomposition dynamics of organic amendments in peat soils are strongly controlled by substrate quality, moisture regime, and microbial accessibility (Elsgaard et al. 2012; Barel et al. 2021; Raczka et al. 2021). Following the 20 cm lowering of the water table in the second year, CO_2_ emissions increased markedly, reflecting enhanced decomposability of organic substrates under improved aeration (Leifeld et al. 2020). A clear hierarchy in cumulative CO_2_ release was observed: paper waste > cereal straw > *Miscanthus* chips > biosolids > biochar, which closely corresponds to substrate lability and carbon availability. Amendments with substantial labile compounds, particularly paper waste and cereal straw, were the most sensitive to drainage. While a high C:N ratio (C:N 255) is generally associated with slower mineralization, the behaviour of paper waste appears influenced by its structural and biochemical composition, rich in cellulose, hemicellulose and lignin, which may increase microbial accessibility under aerobic conditions while providing a substantial pool of labile carbon under anaerobic conditions (Ojewumi et al. 2022). For instance, cumulative CO_2_ emissions from cereal straw increased by 42% under WTL_20_ compared with WTL_0_ (Fig. 1d), consistent with oxygen exposure stimulating microbial oxidation of labile carbohydrates (Hodgkins et al. 2018; Akinbi et al. 2022). Such substrates, which appear relatively stable under saturated conditions, rapidly decompose when aerobic microsites develop. This pattern is further supported by the significant positive correlation between soil DOC and cumulative CO_2_ efflux (*r* = 0.02, *p* < 0.001) except the biosolid treatment, indicating that amendments with higher decomposability accelerate soil organic matter mineralization and carbon loss (Wang et al. 2018; Hu et al. 2024). However, because biosolids decompose rapidly and do not persist in peat soils over multi-year timescales, their long-term influence on CO_2_ emissions increasingly resembles that of the unamended peat, explaining the convergence of biosolids and control treatments despite contrasting initial C:N ratios. Collectively, these findings emphasize and confirm our first hypothesis that the introduction of organic amendments can increase CO_2_ emissions during drainage events, potentially triggering substantial short-term carbon release from peat soils.

In contrast, the Biochar treatment demonstrated a markedly different response. Although CO_2_ emissions from biochar-treated peat also increased under WTL_20_ (from 0.9 t CO_2_ ha^−1^ at WTL_0_ to 2.5 t CO_2_ ha^−1^), the overall flux remained substantially lower than that from other amendments (Fig. 1c, d). This relative stability reflects the inherently recalcitrant nature of biochar carbon and its limited microbial accessibility, even under more aerobic conditions (Bruun et al. 2012; Kuzyakov et al. 2014). The high proportion of stable pyrogenic carbon (SPAC = 22.1–25.2%) and elevated H/C atomic ratio (0.6567) (Additional file 1: Table S3) confirm its long-term chemical stability (Lehmann et al. 2011; Yu et al. 2025). Over the two-year experiment, biochar likely buffered CO_2_ release by protecting native soil organic matter through sorptive stabilization and by moderating oxygen diffusion and microbial activity (Yao et al. 2023; Jeewani et al. 2025b). Moreover, evidence from laboratory studies indicates that biotic aging of biochar leads to surface oxidation and formation of O- and H-containing functional groups, enhancing its interaction with soil minerals and further reducing its degradability (Quan et al. 2020). Thus, while drainage intensifies decomposition of labile substrates, biochar maintains a low and relatively stable CO_2_ emission profile, confirming its potential as a carbon-stabilizing amendment under fluctuating hydrological regimes. Aligning amendment selection with water table management is therefore critical to minimizing emission risks and achieving long-term peatland restoration and climate mitigation goals.

### Methane dynamics and climate trade-offs

Our results clearly demonstrate the dominant influence of water table depth and amendment type on CH_4_ fluxes in agricultural peat soils. Lowering the water table from WTL_0_ to WTL_20_ led to a near-complete suppression of CH_4_ emissions (90–98%), highlighting the strong hydrological control on methanogenesis. This reduction is consistent with global syntheses showing that CH_4_ production declines exponentially once the water table drops below approximately 20 cm, as increased oxygen diffusion inhibits the activity of obligate anaerobic methanogens (Conrad 2020; Boonman et al. 2024). In contrast, sustained CH_4_ emissions under WTL_20_ reflect the persistence of anaerobic microsites within the saturated peat matrix, supporting localized methanogenic activity despite generally oxic conditions (Keiluweit et al. 2017). The significant decline in CH_4_ flux under WTL_20_ therefore represents a favourable outcome for peatland GHG balance, as CH_4_ has a global warming potential approximately 27 times higher than CO_2_ on a 100-year timescale. Consequently, even moderate drainage can yield substantial climate benefits in agricultural peatlands when managed carefully to avoid excessive CO_2_ losses.

Organic amendment-specific responses further illustrate how substrate quality modulates CH_4_ dynamics under differing hydrological regimes. At WTL_0_, labile organic inputs such as paper waste enhanced CH_4_ emissions by stimulating acetolactic methanogenesis through readily available carbon substrates and rapid redox decline (Dyksma et al. 2020; Zhou et al. 2024). These responses are characteristic of low C:N ratio amendments that favour methanogenic pathways over competing anaerobic processes (Dalal et al. 2008; Dean et al. 2018). In contrast, biochar consistently reduced CH_4_ emissions under both saturated and drained conditions, highlighting its mitigation potential. This suppression likely arises from both physical and biochemical mechanisms: biochar’s porous matrix supports colonization by facultative methanotrophs, enhancing CH_4_ oxidation (Wu et al. 2019), while redox-active functional groups (e.g., quinones) inhibit methanogens by competing for electrons or disrupting key metabolic pathways (Nan et al. 2021).

Collectively, these findings identify WTL_20_ as a hydrological threshold that effectively suppresses CH_4_ emissions without fully compromising peat moisture integrity. From a management perspective, maintaining the water table near this level offers an optimal compromise between reducing CH_4_-driven radiative forcing and limiting CO_2_ release from enhanced aerobic decomposition. Such dynamic water table regulation, combined with recalcitrant amendments like biochar, provides a long-term C retention and stabilization in agricultural systems.

### Mechanistic drivers of N_2_O fluxes

In year 2, when the water table was lowered to 20 cm, a pronounced shift in N_2_O emission dynamics was observed across treatments, reflecting strong hydrological control on nitrogen transformation pathways (Yang et al. 2013; Gao et al. 2014). Drainage to WTL_20_ created partially aerobic conditions that promoted coupled nitrification–denitrification processes conditions known to maximize N_2_O production (Liu et al. 2016b; Marsden et al. 2019; Zhao et al. 2025). Under these conditions, inorganically amended treatments exhibited a substantial rise in cumulative N_2_O emissions, with fluxes increasing up to 1.5–2.0 times relative to the first year. This marked increase likely stems from enhanced nitrification under improved oxygen availability, followed by incomplete denitrification in transiently anaerobic microsites, consistent with the “hole-in-the-pipe” conceptual model (Liu et al. 2016b).

By contrast, the Control treatment showed no comparable increase in N_2_O flux after drainage. This lack of response can be attributed to the limited availability of labile carbon and reduced substrate supply for denitrifiers, constraining both nitrification rates and subsequent N_2_O formation. Without sufficient organic carbon to sustain microbial respiration, denitrification may have proceeded more completely to N_2_, or total N turnover may have declined altogether (Zhu et al. 2013; Anderson et al. 2014).

Among the organic amendments, biosolids and cereal straw showed the greatest N_2_O enhancement at WTL_20_, reaching cumulative emissions of approximately 0.014–0.015 t N_2_O-N ha^−1^ by the end of the experiment. Their low C:N ratios and high mineral-N content likely accelerated nitrification and provided ample electron donors for incomplete denitrification, enhancing gaseous N losses. Biochar and *Miscanthus* chips also showed modest increases, yet their cumulative emissions remained lower than those of biosolids and straw. Biochar application reduced N_2_O emissions in our study. This effect is consistent with previous reports that biochar’s porous structure can improve soil aeration and water retention, potentially stabilizing oxygen availability and influencing nitrification- denitrification dynamics. Its physicochemical properties, including high carbon stability and redox-active surfaces, may contribute to enhanced nitrogen retention and reduced N_2_O production. Microcosm studies have further suggested that biochar can alter microbial community composition, favoring populations associated with lower N_2_O emissions. Collectively, these properties likely underpin the restrained N_2_O fluxes observed, although the exact mechanisms were not directly measured in this experiment (Cayuela et al. 2013, 2014). Overall, these results highlight that WTL_20_ represents a hydrological threshold conducive to elevated N_2_O formation, particularly in systems receiving low C:N amendments. Conversely, treatments with more recalcitrant carbon sources especially biochar demonstrated improved N_2_O mitigation potential. Biochar application reduced N_2_O emissions, likely due to its unique physicochemical properties. Its porous structure improves soil aeration and water retention, promoting more stable oxygen conditions that influence coupled nitrification–denitrification processes (Zhou et al. 2025). Additionally, biochar’s redox-buffering capacity helps stabilize soil electron acceptor availability, further modulating microbial N transformations. Recent meta-analyses and mechanistic studies have demonstrated that biochar regulates denitrification pathways by increasing the abundance of *nosZ*-harboring microorganisms and enhancing the *nosZ*/(*nirS* + *nirK*) ratio, thereby promoting the complete reduction of N_2_O to N_2_ (Zhong et al. 2025; Zhou et al. 2025). Moreover, microcosm experiments show that biochar can shift microbial community composition toward denitrifiers with lower N_2_O production potential, consistent with the emission reductions observed in our study (Lin et al. 2024). Thus, integrating moderate water-table management with stable carbon amendments may offer a practical strategy to minimize total GHG emissions while maintaining nutrient cycling in managed peat soils.

### Net greenhouse gas emissions (CO_2_eq) under contrasting water table regimes

This study highlights the strong interactive influence of hydrological regime and organic amendment type on the overall GHG balance of agricultural peat soils. Although CO_2_ emissions increased at WTL_20_ during year 2, the total CO_2_eq emission was markedly lower than that observed under saturated conditions (WTL_0_) in the first year (Fig. 4). This apparent paradox is explained by the suppression of CH_4_ emissions following drainage, as methanogenesis is highly sensitive to redox conditions and rapidly declines once oxygen penetrates the upper peat layer. Because CH_4_ possesses a global warming potential approximately 27 times greater than CO_2_ (IPCC Sixth Assessment Report 2023), even moderate reductions in CH_4_ flux can offset significant increases in CO_2_ emissions. In contrast, the near-saturated WTL_0_ treatment strongly favoured anaerobic processes, leading to enhanced CH_4_ production, a pattern consistent with numerous peatland studies demonstrating enhanced methanogenesis under waterlogged conditions with abundant labile carbon (Yrjälä et al. 2011; Boonman et al. 2024). At WTL_20_, CH_4_ emissions declined by more than half, while CO_2_ and N_2_O together accounted for approximately 95% of total CO_2_eq emissions, reflecting a shift toward aerobic decomposition and partial nitrification–denitrification activity in the aerated peat profile (Mäkiranta et al. 2012; Liu et al. 2016a; Zhao and Zhuang 2024). From a climate mitigation perspective, these findings suggest that maintaining a moderate water table depth at approximately 20 cm below the surface provides a more favourable balance between limiting CH_4_ emissions and avoiding extensive peat oxidation associated with deeper drainage. Similar thresholds have been identified in both field and modelling studies, where partial drainage reduced total GHG fluxes relative to fully saturated or deeply drained peatlands (Evans et al. 2021b; Kalhori et al. 2024; Yang et al. 2025). Therefore, controlled water table management coupled with the use of stable organic amendments such as biochar can optimize GHG outcomes by suppressing high-GWP CH_4_ emissions while minimizing long-term CO_2_ losses from peat decomposition.

Among the organic amendments across the two experimental years, the transition from saturated (WTL_0_) to moderately drained (WTL_20_) conditions markedly altered the composition and magnitude of CO_2_eq emissions (Figs. 3, 4). In year 1, at WTL_0_, CH_4_ was the dominant, accounting for more than half of total CO_2_eq emissions due to sustained methanogenic activity within the anaerobic peat layer. This response is consistent with findings that methanogenesis is primarily confined to the upper 10–30 cm of the peat profile, where methanogenic archaea inhabit microsites rich in labile carbon and poor in terminal electron acceptors (Yrjälä et al. 2011; Bridgham et al. 2013). The saturated conditions at WTL_0_ maintained a highly reducing environment that favoured acetoclastic and hydrogenotrophic methanogenesis (Conrad 2020), resulting in substantial CH_4_ release. However, lowering the water table to 20 cm in year 2 almost completely suppressed CH_4_ emissions, as increased oxygen penetration into the upper peat inhibited obligate anaerobic methanogens and stimulated methanotrophic CH_4_ oxidation (Freeman et al. 2022; Boonman et al. 2024). Although CO_2_ fluxes increased under these more aerobic conditions, the overall CO_2_eq emissions were lower than at WTL_0_, indicating that CH_4_ suppression outweighed the rise in CO_2_ production.

The more labile amendments, cereal straw and *Miscanthus* chips generated the highest total CO_2_eq emissions at WTL_20_, reflecting their labile carbon composition and low C:N ratios, which accelerated both aerobic decomposition and coupled nitrification denitrification processes (Leifeld et al. 2020; Gu et al. 2022). Such amendments enhanced substrate availability for microbial metabolism, amplifying CO_2_ and N_2_O formation under drained conditions.

In contrast, biochar consistently resulted in the lowest CO_2_eq emissions across both hydrological regimes, highlighting its resilience and mitigation potential. At WTL_0_, biochar-treated soils emitted approximately 8.6 t CO_2_eq ha^−1^ yr^−1^), while under WTL_20_, emissions were slightly higher (12.2 t CO_2_eq ha^−1^ yr^−1^), representing only a 1.4-fold increase despite greater aeration. This stable performance reflects multifunctional capacity of biochar: it enhances CH_4_ oxidation through the promotion of methanotrophic colonization, adsorbs reactive nitrogen intermediates to limit N_2_O production, and stabilizes organic carbon via its aromatic structure and high surface area (Mukherjee and Lal 2013; Wang et al. 2023). These results highlight that GHG dynamics in managed peatlands depend on both hydrological control and substrate chemistry. An integrated strategy combining water table regulation (at WTL_20_) with chemically stable amendments such as biochar offers a promising path toward minimizing CO_2_eq emissions, maintaining peat carbon stocks, and enhancing the long-term climate resilience of agricultural peatlands (Fig. 5). Overall, the results indicate that lowering the water table substantially altered the redox conditions and microbial activity within the peat, leading to enhanced CO_2_ emissions and reduced CH_4_ production, thereby demonstrating the critical role of water table position in regulating GHG dynamics in peatlands.

## Conclusion

This study confirms that water table depth is the dominant driver of GHG dynamics in agricultural peat soils. Over two consecutive years, contrasting hydrological regimes revealed that drainage to 20 cm depth enhanced aerobic decomposition and increased CO_2_ emissions but simultaneously suppressed CH_4_ fluxes to negligible levels, resulting in lower overall CO_2_eq emissions compared to the CH_4_-dominated fluxes under WTL_0_ conditions. Given the 27-fold higher global warming potential of CH_4_ than CO_2_, maintaining a moderately lowered water table presents a more climate-efficient strategy than full saturation. Organic amendment quality further modulated these responses. Labile, low C: N amendments such as cereal straw and biosolids stimulated microbial mineralization and elevated CO_2_ and N_2_O emissions, particularly under drainage, increasing total CO_2_eq fluxes. In contrast, biochar consistently exhibited the lowest net GHG emissions across both hydrological regimes, (19.77 t CO_2_eq ha^−1^ yr^−1)^ lower than other amendments and controls, owing to its aromatic stability, porosity, and redox-buffering properties that promote CH_4_ oxidation and stabilize soil carbon. Overall, alternating WTL_0_ and WTL_20_ water table management combined with stable, recalcitrant amendments such as biochar represents a practical way to mitigate GHG emissions in lowland peat soils; however, future field-based studies incorporating vegetation and variable hydrological regimes are needed to fully assess their agronomic relevance.

## Supplementary Information

**Additional file 1.**

## Data Availability

Data will be made available on request.
