# Supplementary material for: Biochar mitigates the peatland GHG dilemma under contrasting water table regimes: phase-dependent responses of CO2 and CH4 over a two-year study
Source: Biochar. 2026 Apr 21;8(1):93. doi: 10.1007/s42773-026-00610-2 (PMC13095913; doi:10.1007/s42773-026-00610-2)
Supplement: Supplementary file 1 — Additional file 1. [file 42773_2026_610_MOESM1_ESM.docx]

**Biochar mitigates the peatland GHG dilemma under contrasting water table regimes: Phase-dependent responses of CO₂ and CH₄ over a two-year study**

Peduruhewa H. Jeewani^a*^, Jennifer M. Rhymes^b^, Chris D. Evans^b^, Davey L. Jones^a^, David R. Chadwick^a^

^a^ School of Environmental and Natural Sciences, Bangor University, Bangor, Gwynedd, LL57 2UW, UK

^b^ UK Centre for Ecology & Hydrology, Bangor, Gwynedd, LL57 2UW, UK

^*^Corresponding author: Peduruhewa H. Jeewani

Corresponding Author Address: School of Environmental and Natural Sciences, Bangor University, Bangor, Gwynedd, LL57 2UW, UK

Corresponding Author Email: j.hemamali@bangor.ac.uk

**(B)**


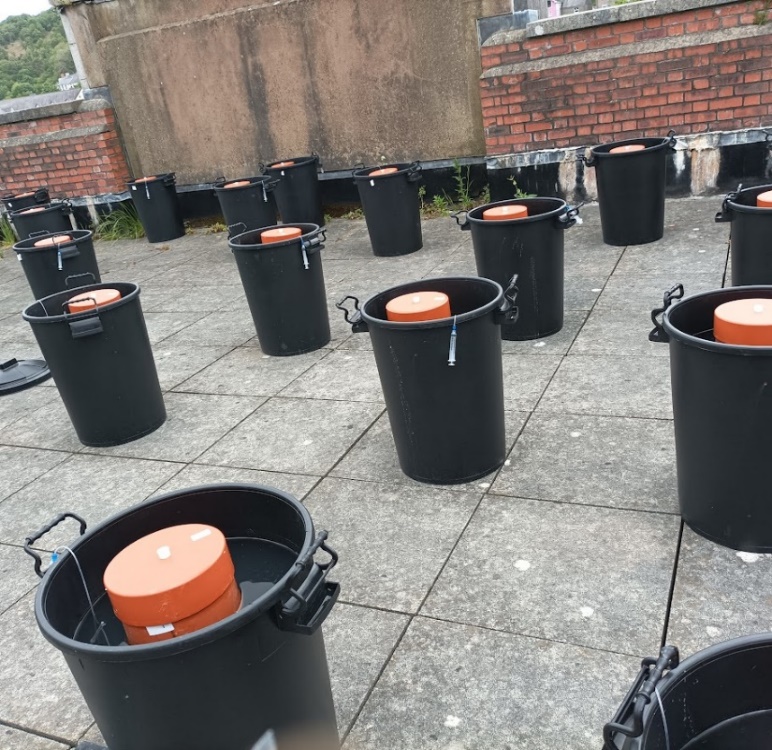

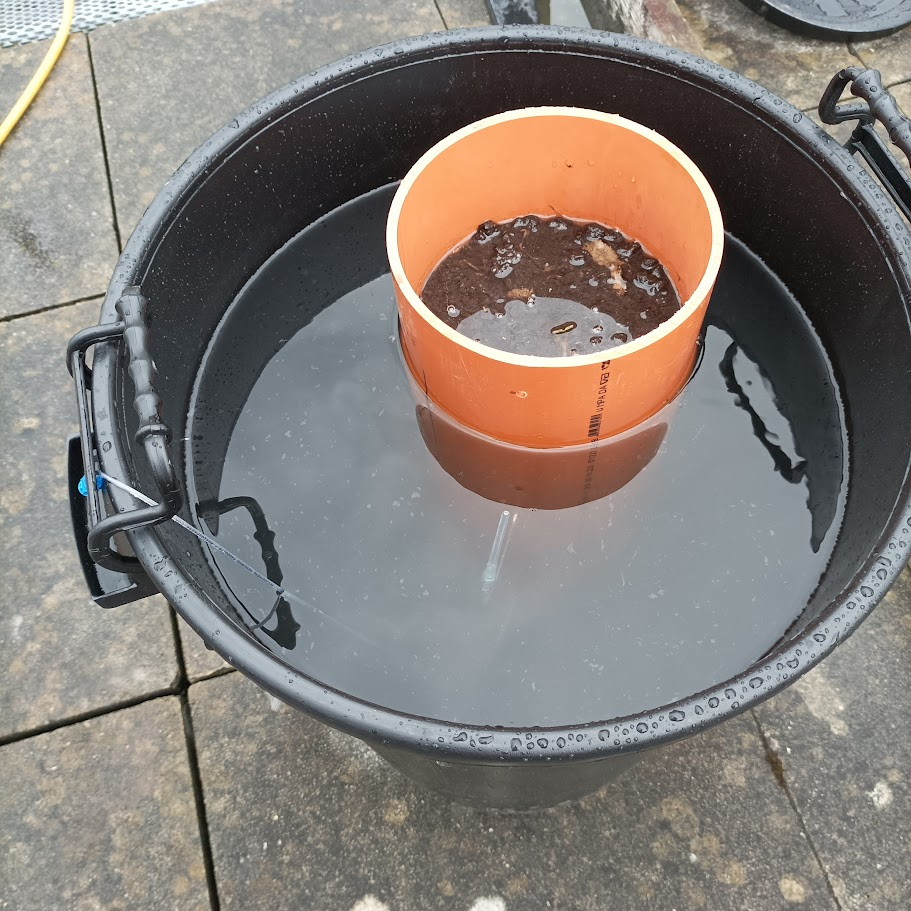


**(A)**


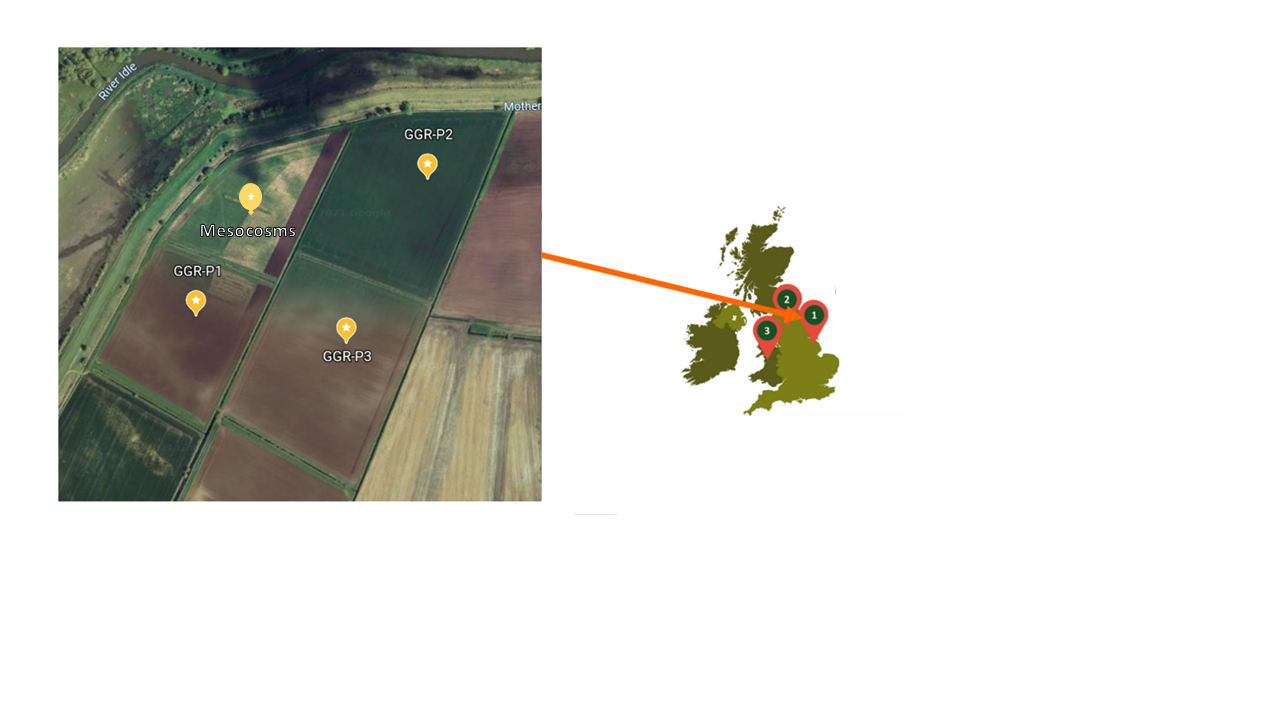

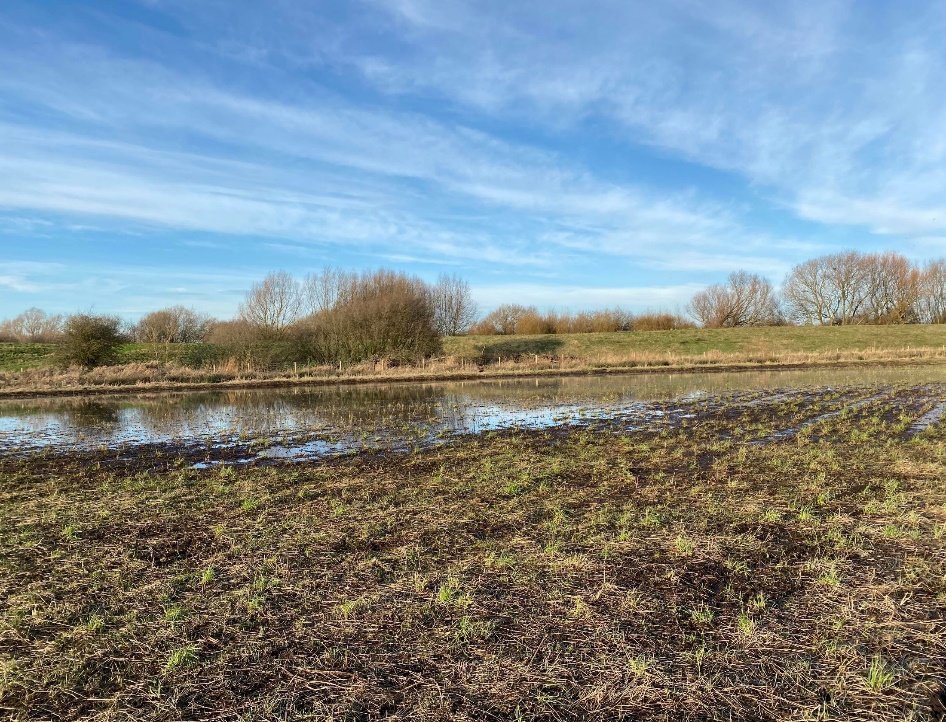

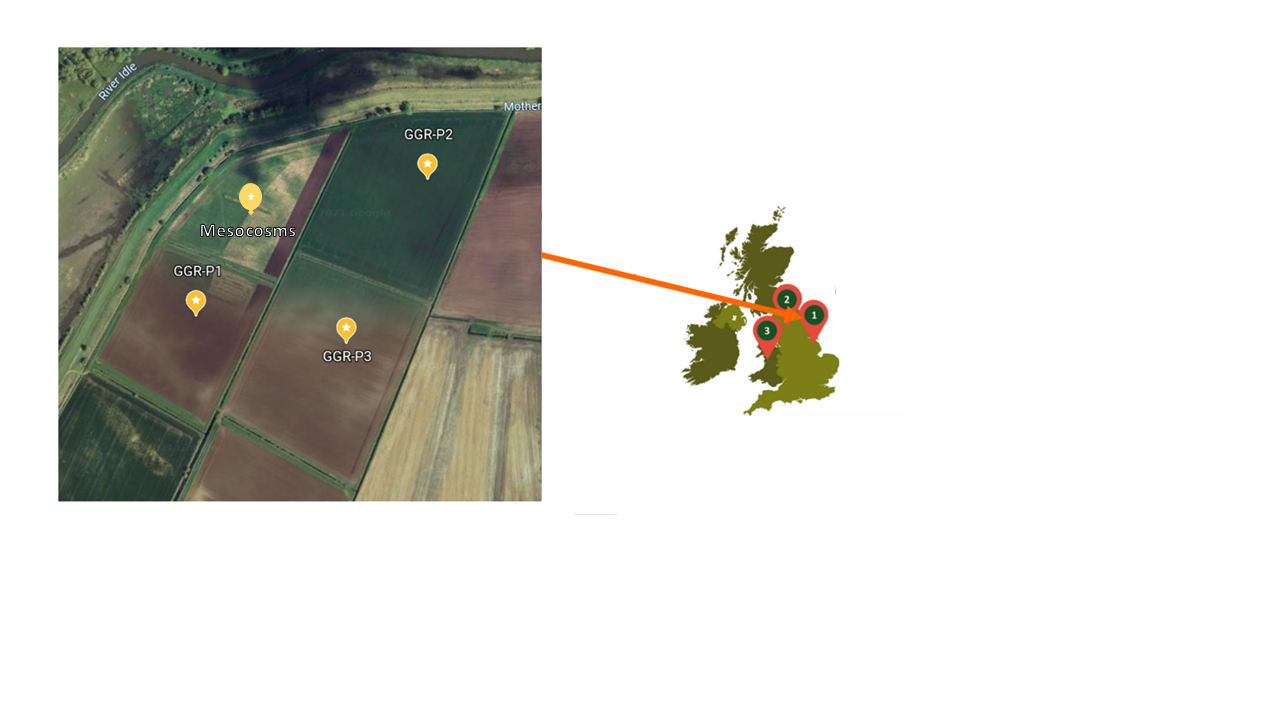

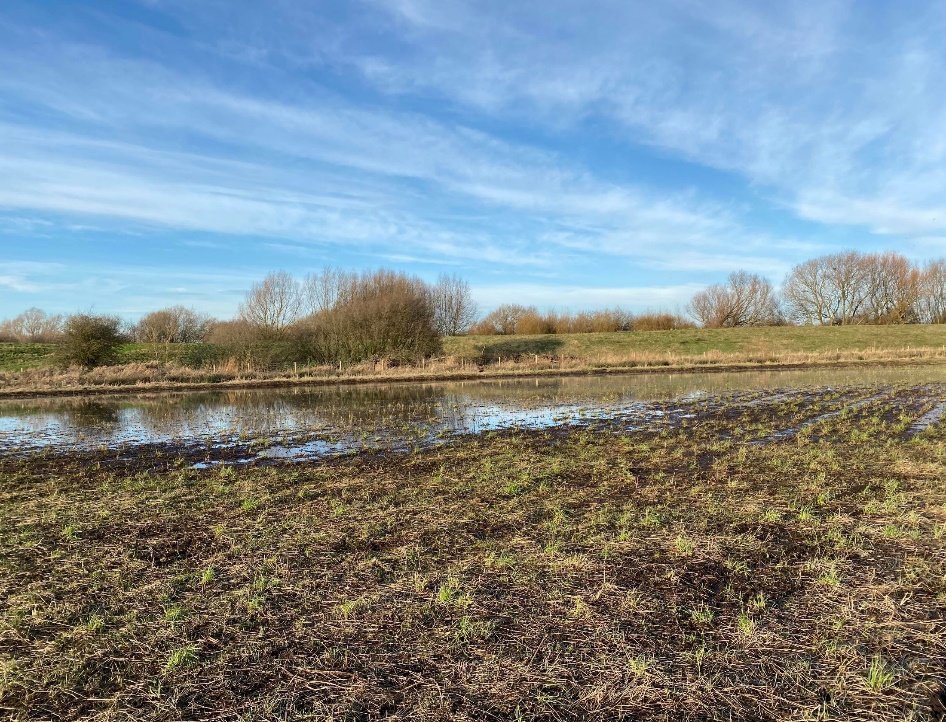

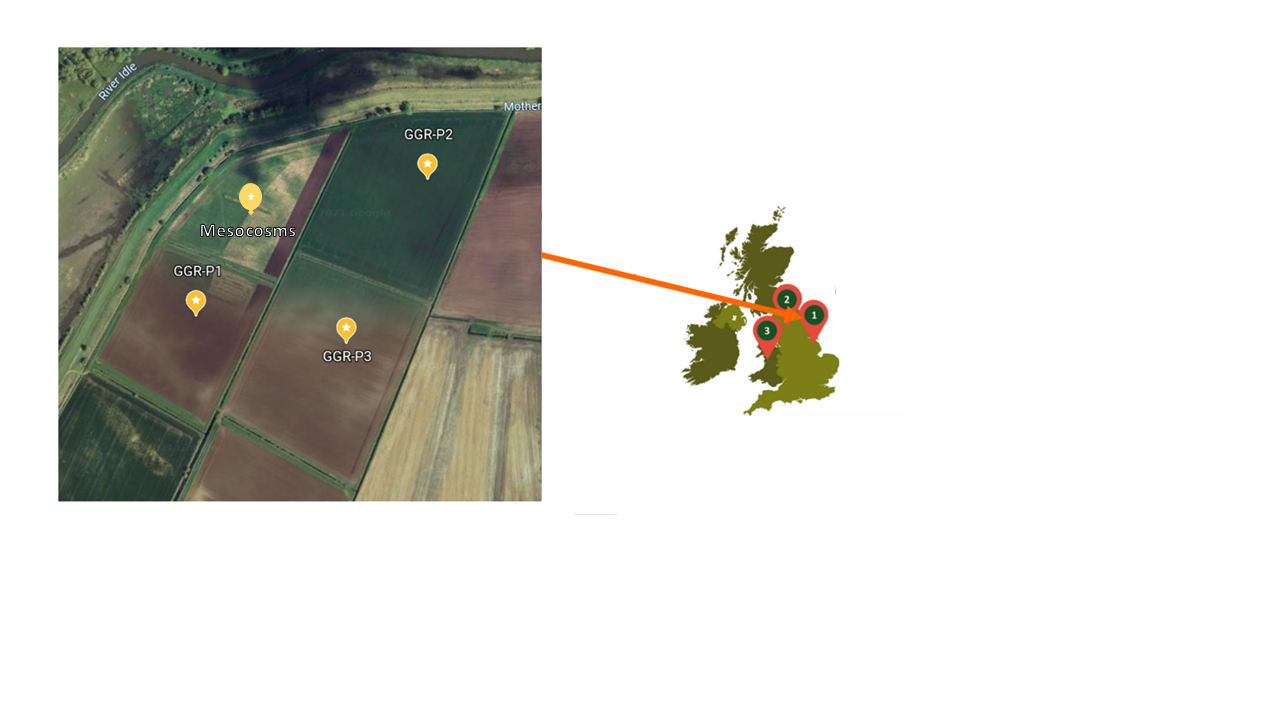


**(D)**

**(C)**

**Fig. S1:** Agricultural peat land sampling site and soil mesocosm setup (A and B). Intact peat soil mesocosms were placed into an outer container with drainage holes drilled to maintain the water table level at a specific height, e.g. level with the soil surface at 0 cm (C). Headspace greenhouse gas sampling from the mesocosms after fitting a gas-tight lid (D).


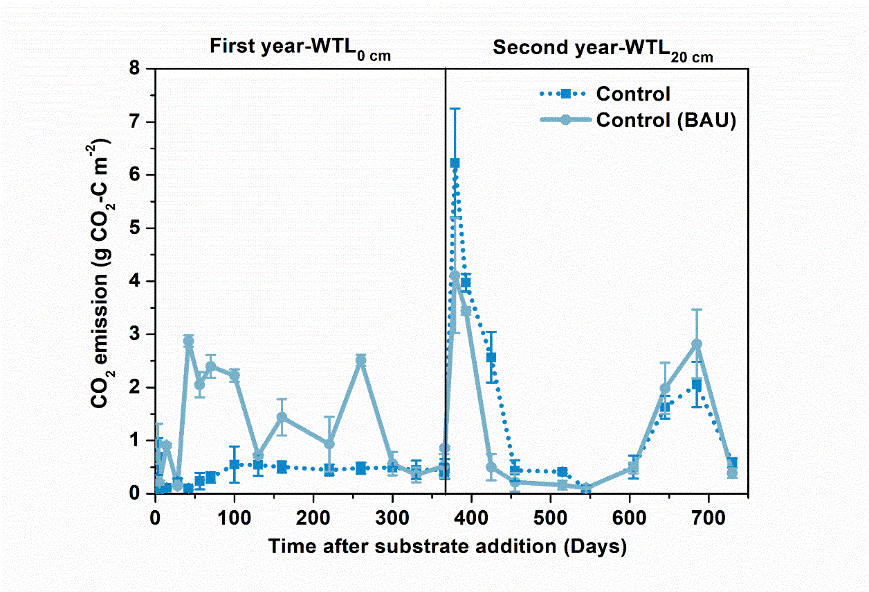

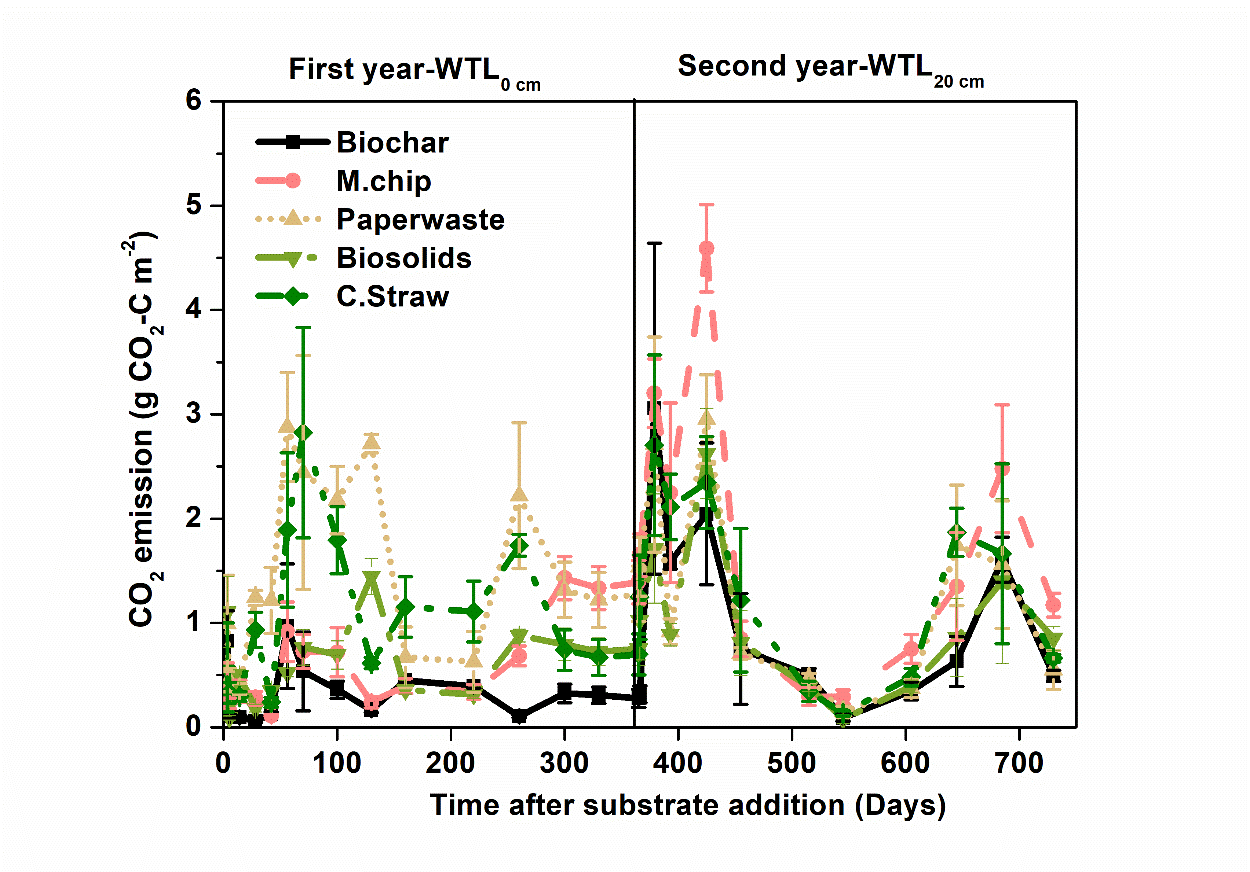

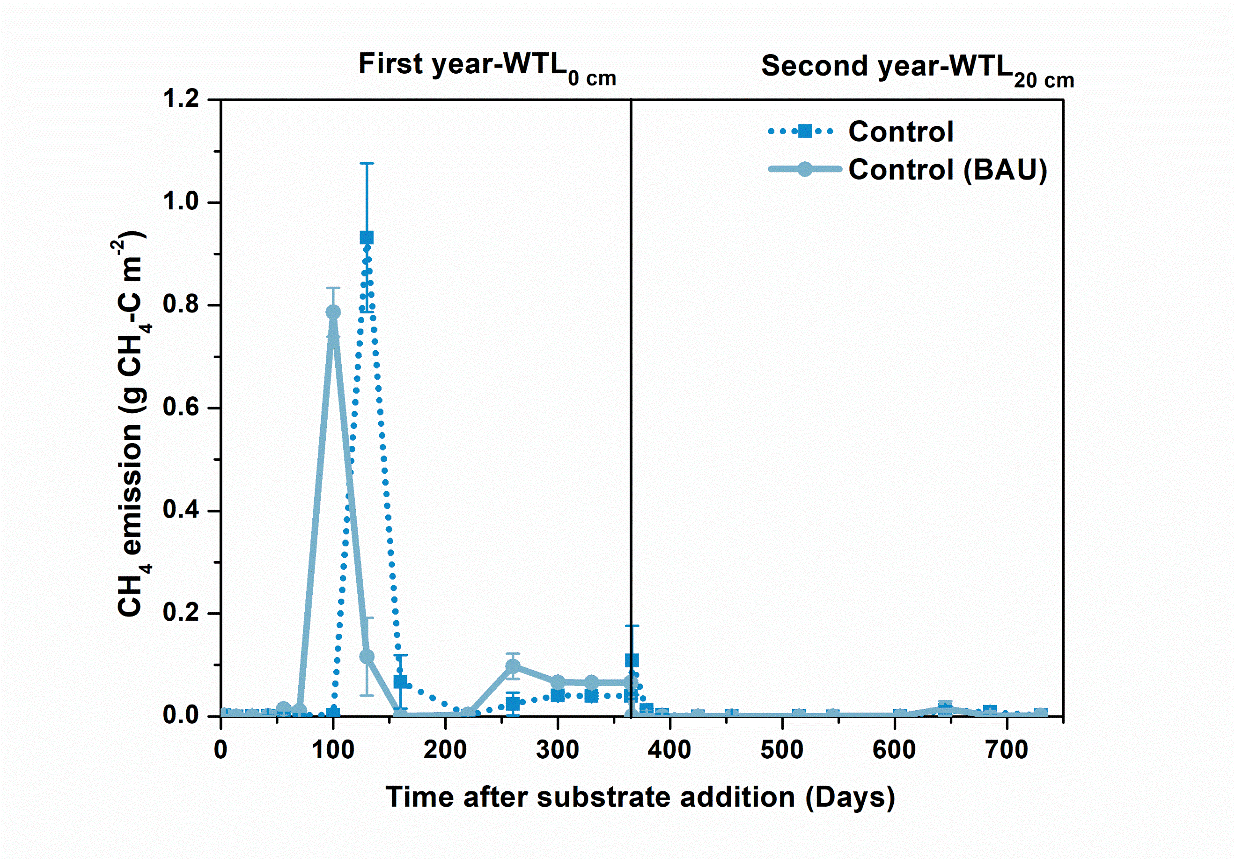

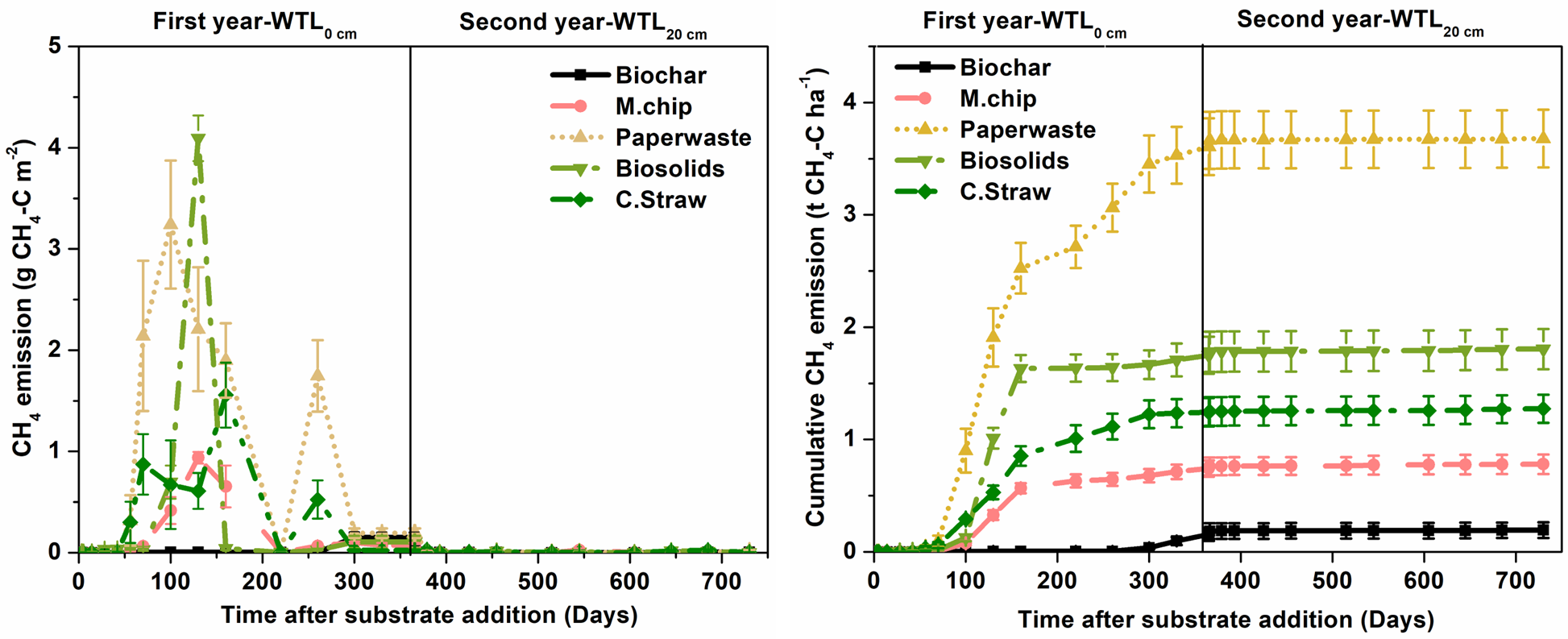

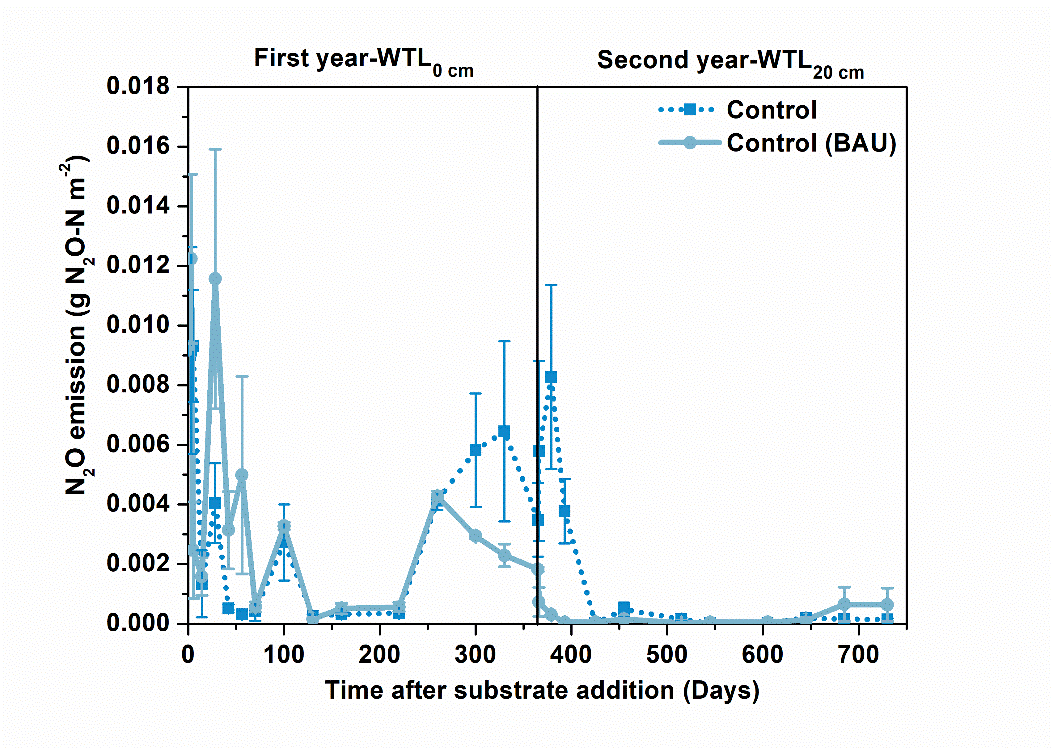

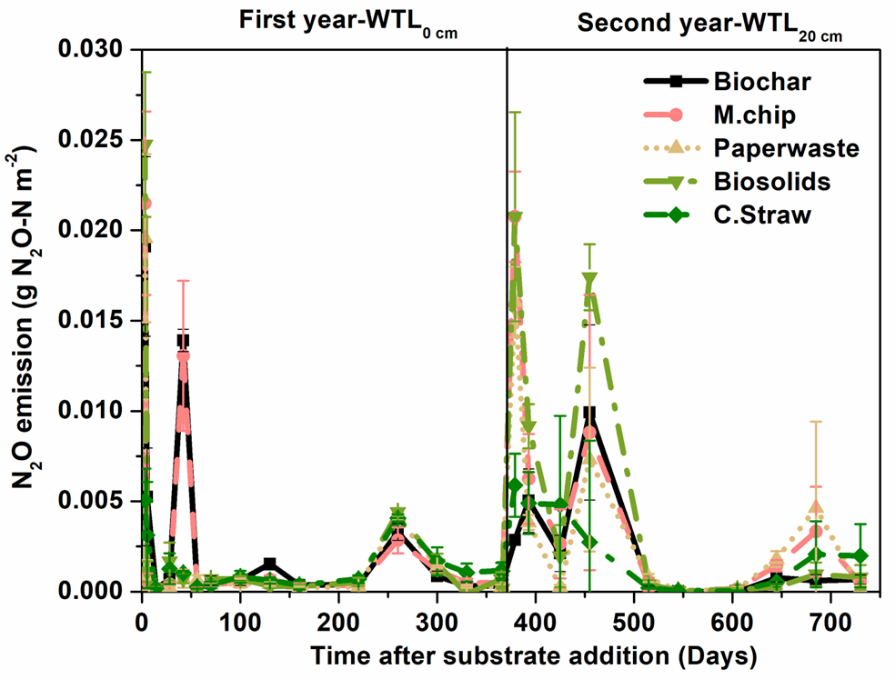


**Fig. S2.** Temporal dynamics of GHG fluxes (CO_2_, CH_4_ and N_2_O) under rewetted (Year 1) and drained (Year 2) conditions across soil amendments
Cumulative CO_2_ emissions from controls (a) and organic amendments (b) at both water table levels. The organic amendments included *Miscanthus* biochar (Biochar), *Miscanthus* chips (M.chip), paper waste, biosolids and cereal straw (C.Straw). The water table level was at 0 cm (WTL_0_, saturated) in year 1 and at 20 cm (WTL_20_, moderately drained) in year 2. The water table depth of the BAU Control was at 40 cm (WTL_40_) throughout the two-year experimental period. Values represent mean ± standard errors (*n* = 4). Note the different y-axis scales for all graphs.





**Fig. S3** Effect of organic amendment addition on the carbon balance in an agricultural peat soil over two years. The organic amendments included *Miscanthus* biochar (Biochar), *Miscanthus* chips (M.chip), paper waste, biosolids and barley straw (C.Straw). The Control treatments included a water table depth of 0 cm (WTL_0_, saturated) in year 1 that transitioned to a water table depth of 20 cm (WTL_20_, moderately drained) in year 2. The water table depth of the BAU Control was 40 cm (WTL_40_) throughout the two-year experimental period. Values represent mean ± standard errors (*n* = 4).


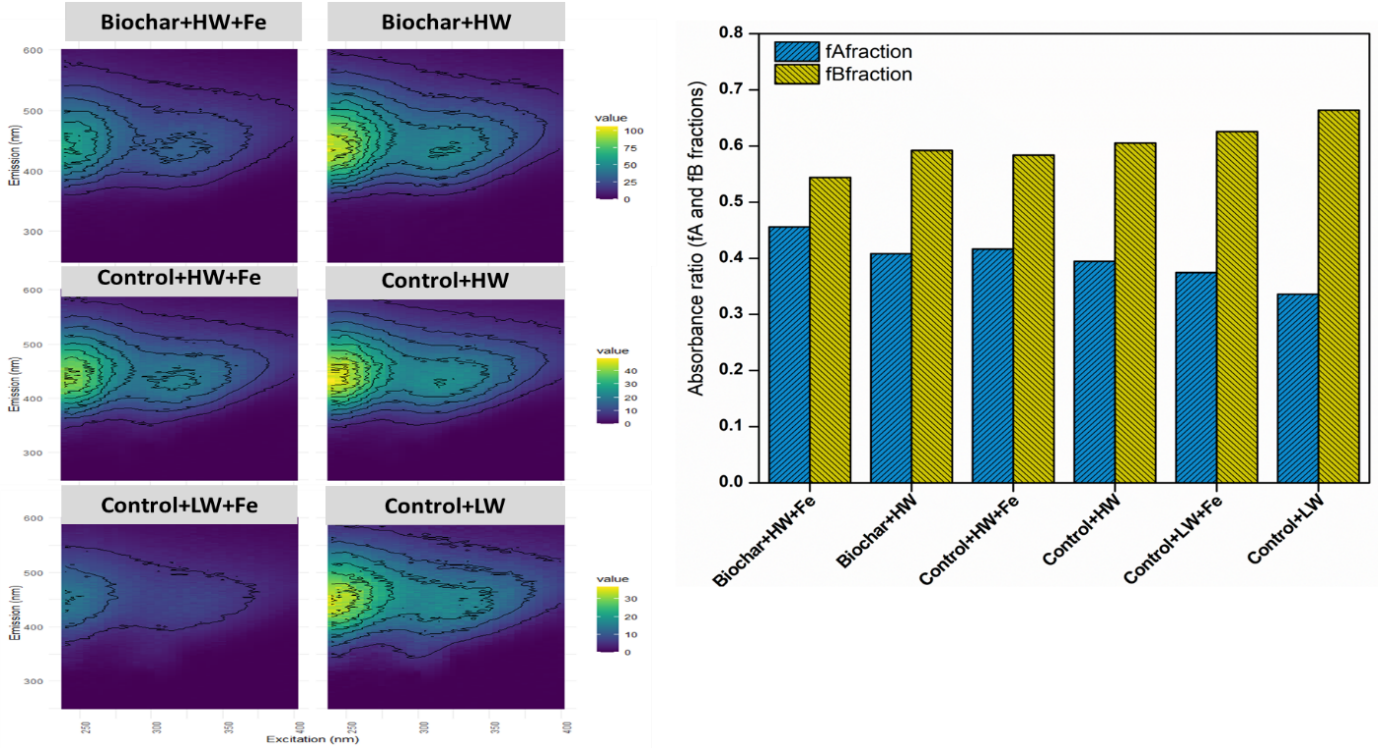

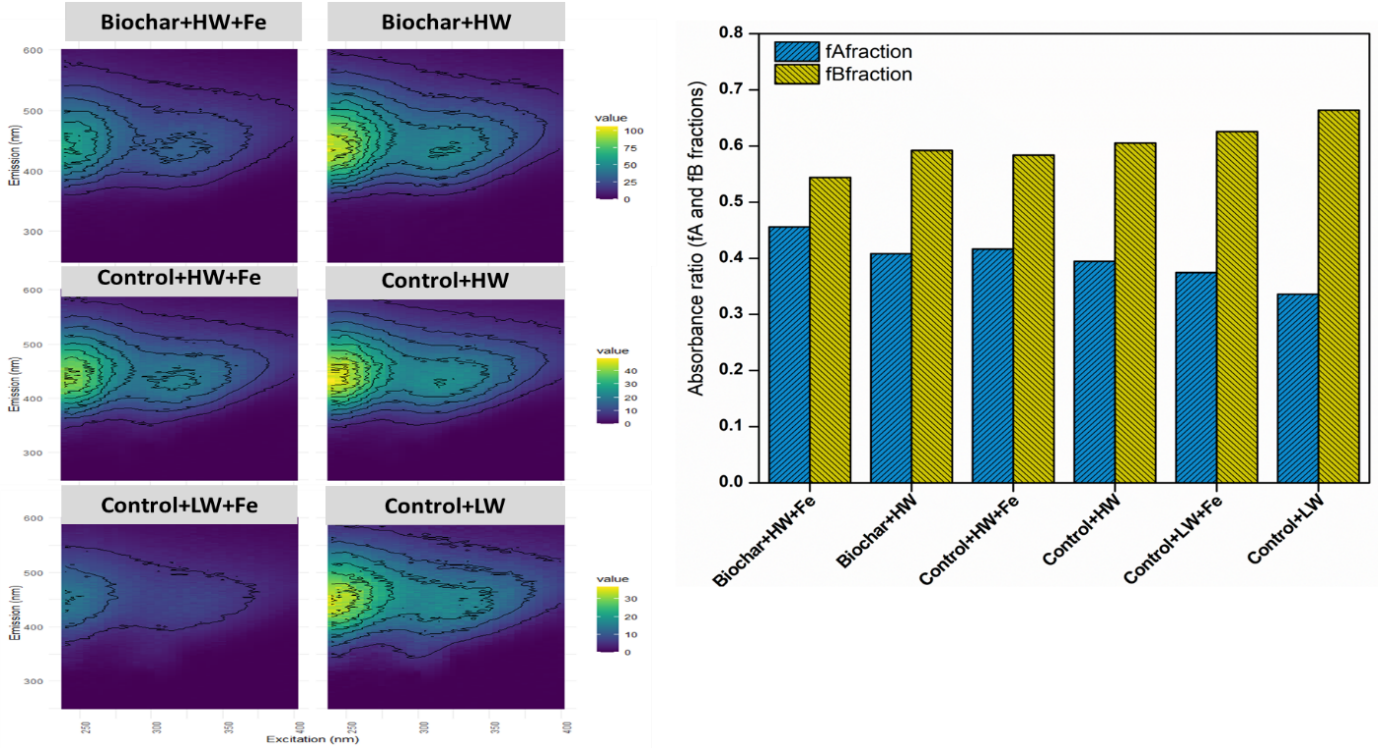


**Biochar**

**Control WTL 0 cm 0cm**

**Control WTL 40 cm 0cm**

**Fig. S4.** Effect of biochar amendment dissolved organic matter (DOM) included biochar (pyrolysed *Miscanthus giganteus* chip). The Control treatments included a water table at 0 cm (WTL_0_, saturated) in year 1 that transitioned to a water table depth of 20 cm (WTL_20_, moderately drained) in year 2. The water table depth of the BAU Control was 40 cm (WTL_40_) throughout the two-year experimental period. Values represent mean ± standard errors (*n* = 4).


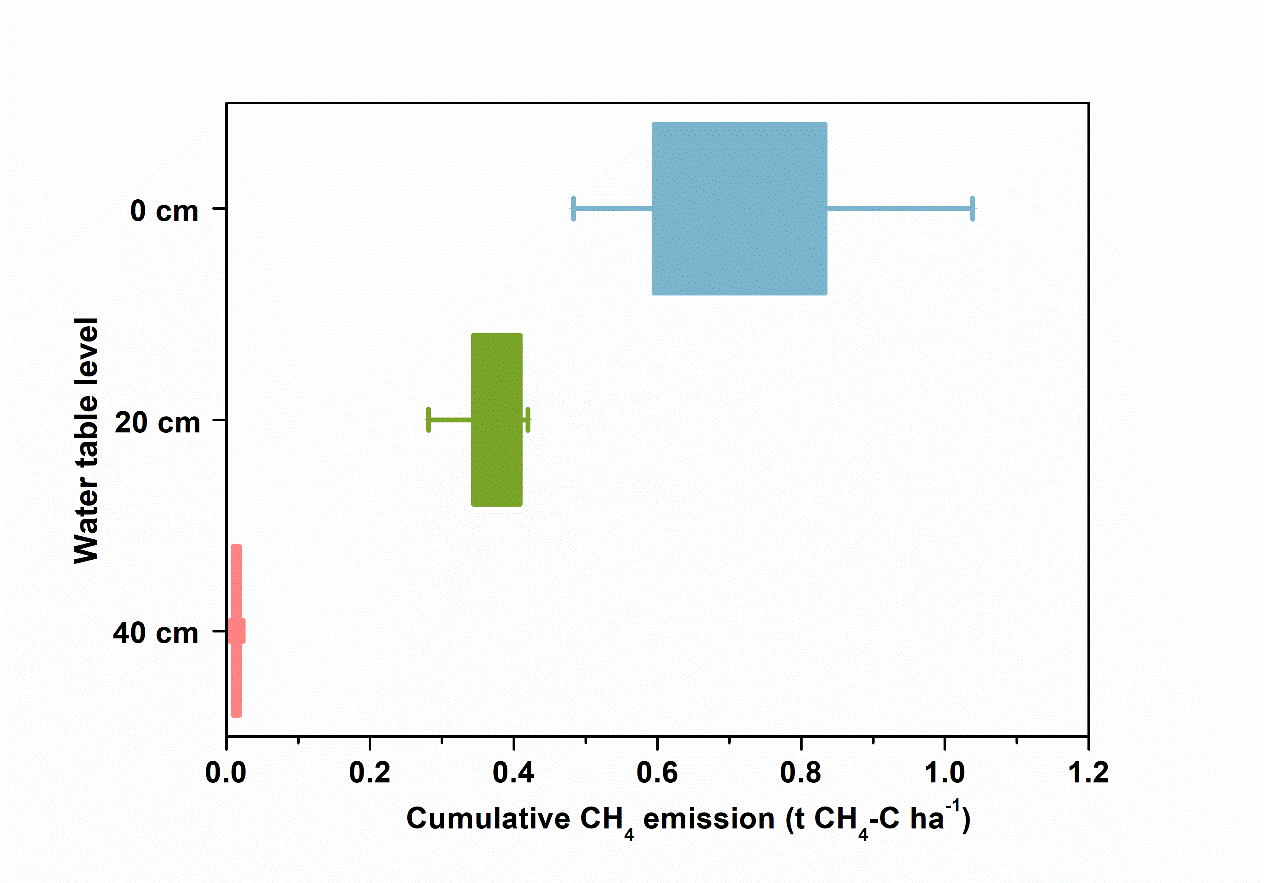

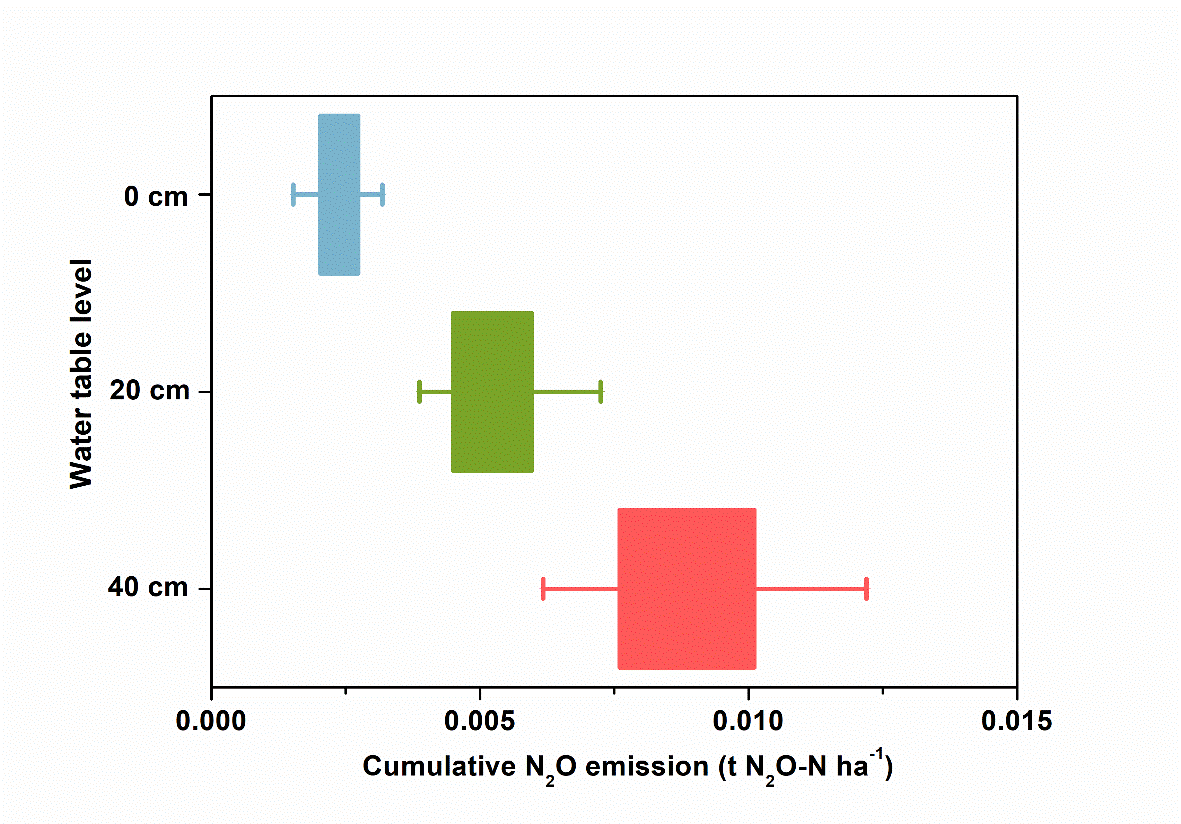

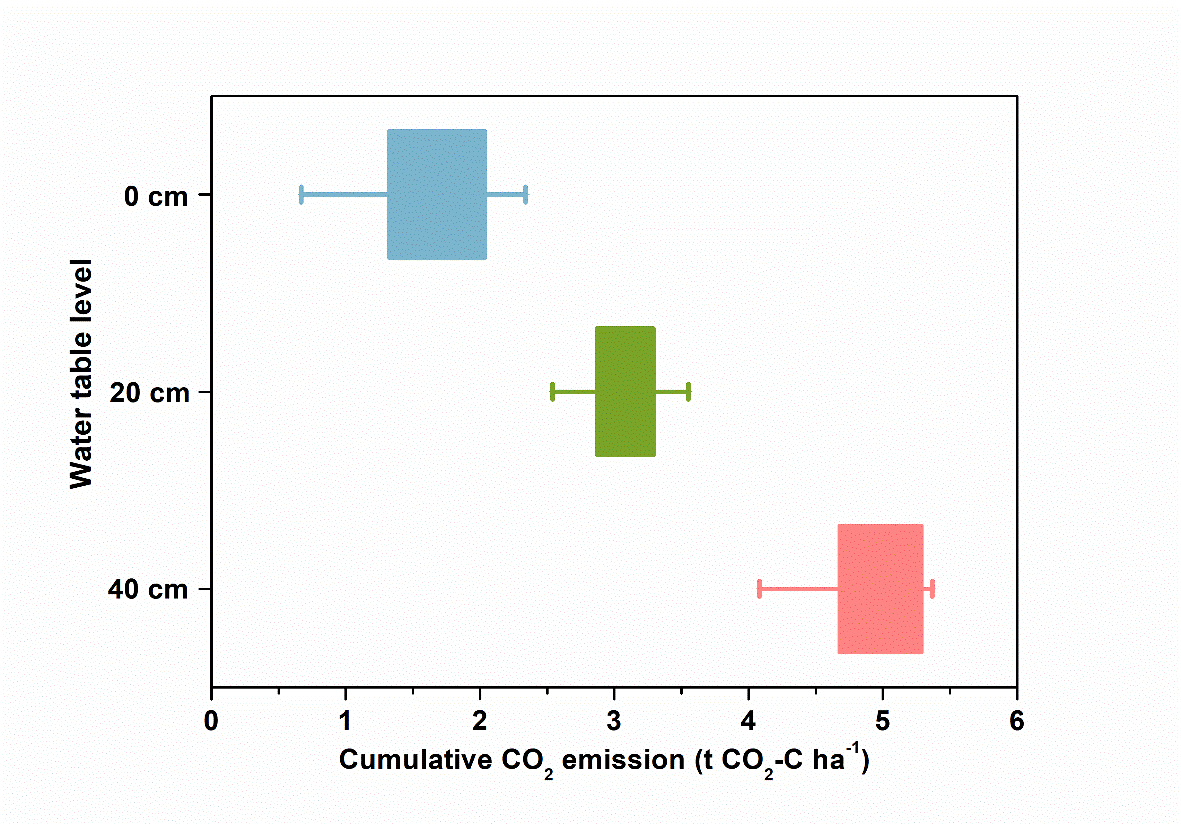


**(a)**

**(b)**

**(c)**

**Fig. S5** Relationship between water table levels (WTL) and cumulative greenhouse gas emissions of Control treatments (a, b and c). The water table level was at the soil surface (0 cm; WTL_0_, saturated) in year 1 and at 20 cm (WTL_20_, moderately drained) in year 2. The water table depth for the BAU Control was 40 cm (WTL_40_) throughout the two-year experimental period. Values represent mean ± standard errors (*n* = 4). Note different x-axis scales for panels a, b and c.

**Table S1.** Physicochemical properties of soil used in the experiments after 2 years. Values represent means ± standard errors (n = 4). Abbreviation: EC, electrical conductivity; DOC; Dissolved organic carbon, TDN total dissolved nitrogen, where applicable, the data are expressed on a dry weight basis.

| **Treatments** | **pH** | **EC**  **(μS cm^−1^)** | **DOC**  **(mg L^-1^)** | **TDN**  **(mg L^-1^)** |
| --- | --- | --- | --- | --- |
| **Miscanthus biochar** | 6.47±0.05 | 71.85±6.2 | 57.61±4.1 | 7.68±0.6 |
| **Miscanthus chip** | 6.36±0.09 | 54.72±6.3 | 33.45±7.1 | 5.28±0.3 |
| **Paper waste** | 6.22±0.08 | 66.52±8.5 | 49.28±7.3 | 6.43±0.4 |
| **Biosolids** | 6.5±0.06 | 88.05±5.2 | 49.39±5.7 | 7.53±1.8 |
| **Cereal straw** | 5.99±0.02 | 276.7±16.7 | 22.38±0.7 | 11.62±0.4 |
| **Control** | 6.40±0.01 | 97.57±12.6 | 39.76±2.6 | 6.57±0.7 |
| **Control (BAU)** | 6.44±0.04 | 58.27±7.1 | 17.30±2.1 | 3.06±0.2 |
|  |  |  |  |  |

**Table S2.** Physicochemical properties of the initial soil and amendments used in the experiments. Values represent means ± standard errors (*n* = 4). Abbreviation: C, carbon; N, nitrogen; SOM, Soil organic matter content; EC, electrical conductivity; BD, bulk density. Where applicable, the data are expressed on a dry weight basis (Jeewani et al., 2025).

| **Properties** | **Soil** | | | ***Miscanthus***  **biochar** | ***Miscanthus* chip** | **Paper**  **waste** | **Barley**  **straw** | **Biosolids** |
| --- | --- | --- | --- | --- | --- | --- | --- | --- |
|  | **0-20 cm** | **10-30 cm** | **30-50 cm** |  |  |  |  |  |
| **Total C (%)** | 27.6±2.6 | 27.6±1.7 | 24.7±0.9 | 79.3±0.9 | 47.4±0.5 | 46.8±0.81 | 43.7±0.5 | 36.45±30 |
| **Total N (%)** | 1.81±0.45 | 1.81±0.13 | 1.80±0.08 | 0.41±0.01 | 0.49±0.07 | 0.75±0.07 | 0.68±0.02 | 3.66±6 |
| **C:N ratio** | 16.0±4.8 | 15.2±1.3 | 13.7±0.9 | 259±14 | 97.9±16 | 62.8±7.3 | 63.5±2.0 | 9.95±0.03 |
| **SOM (%)** | 45.22±2.6 | 33.05±5.6 | 20.79±1.9 | - | - | - | - | - |
| **pH (H_2_O)** | 6.54±0.05 | 6.65±0.04 | 6.54±0.04 | 5.6± 0.61 | 6.9±0.85 | 5.19±0.52 | - |  |
| **EC (μS cm^−1^)** | 193±4 | 186±4 | 199±9 | - | - | - | - | - |
| **BD (g cm^-3^)** | 0.52±0.05 | 0.55±0.03 | 0.53±0.03 | - | - | - | - | - |
| **NO_3_^-^ (mg N L^-1^)** | 4.05±0.29 | 3.52±0.36 | 3.54±0.29 | 0.15±0.01 | 0.11±0.02 | 0.3±0.03 | 5.84±0.98 | 1.7 ±0.2 |
| **NH_4_^+^ (mg N L^-1^)** | 4.48±0.22 | 2.68±0.44 | 4.65±0.58 | 0.25±0.08 | 2.87±0.56 | 1.02±0.08 | 3.79±1.02 | 218±0.15.6 |
| **SO_4_^2-^ (mg S L^-1^)** | 1.35±0.42 | 1.29±0.34 | 1.40±0.30 | - | - | - | - | - |
| **PO_4_^3-^ (mg P L^-1^)** | 1.05±0.11 | 1.23±0.34 | 0.98±0.19 | 3.33±0.98 | 2.26±0.8 | 3.57±0.9 | 4.03±0.89 | 2.32±0.23 |

**Table S3.** Characteristics of the Miscanthus-derived biochar. The biochar assessment for atomic H/C ratio and the fraction of stable polyaromatic carbon (SPAC) determined by hydropyrolysis (Hypy test)

| Chemical properties | Value |
| --- | --- |
| Carbon (%) | 73.31 |
| Hydrogen (%) | 4.07 |
| Nitrogen (%) | 0.35 |
| Atomic H/C | 0.66 |
| SPAC (%) | 23.82 |
| EC (µS cm^-1^) | 51.2 |
| pH | 5.9 |

**Table S4. Pearson correlation coefficients (r) between soil properties and cumulative GHG fluxes**

Significance levels: *** p < 0.001, ** p < 0.01, * p < 0.05, † p < 0.10.

| **Variable** | **DOC** | **TDN** | **SOM** | **MAOM** | **pH** | **EC** | **CO₂** | **CH₄** | **N₂O** |
| --- | --- | --- | --- | --- | --- | --- | --- | --- | --- |
| **DOC** | — | 0.51** (0.006) | −0.01 (0.94) | −0.27 (0.159) | 0.10 (0.600) | −0.28 (0.154) | 0.02*** (<0.001) | −0.32 (0.094) | 0.25 (0.199) |
| **TDN** |  | — | 0.16 (0.413) | −0.17 (0.376) | 0.23 (0.234) | 0.12 (0.527) | 0.01 (0.945) | −0.37† (0.051) | 0.10 (0.629) |
| **SOM** |  |  | — | −0.63*** (<0.001) | −0.09 (0.634) | −0.43* (0.023) | −0.02 (0.936) | 0.01 (0.958) | −0.24 (0.211) |
| **MAOM** |  |  |  | — | 0.26 (0.189) | 0.36 (0.060) | 0.21 (0.289) | 0.26 (0.175) | 0.16 (0.428) |
| **pH** |  |  |  |  | — | 0.23 (0.235) | −0.13 (0.518) | −0.14 (0.482) | 0.29 (0.141) |
| **EC** |  |  |  |  |  | — | −0.21 (0.282) | −0.14 (0.468) | −0.05 (0.791) |
| **CO₂** |  |  |  |  |  |  | — | 0.33 (0.091) | 0.04 (0.852) |
| **CH₄** |  |  |  |  |  |  |  | — | 0.07 (0.741) |
| **N₂O** |  |  |  |  |  |  |  |  | — |

**Table S5. Multiple linear regression explaining cumulative CO₂ emissions**

Values are regression coefficients (β ± SE).
Significance levels: *** p < 0.001, ** p < 0.01, * p < 0.05.

| **Predictor** | **Estimate (β)** | **Std. Error** | **t value** | **p value** |
| --- | --- | --- | --- | --- |
| Intercept | 2202.36 | 2283.14 | 0.97 | 0.346 |
| DOC | 0.11 | 4.16 | 0.03 | 0.980 |
| TDN | 21.18 | 35.40 | 0.60 | 0.556 |
| SOM | 601.16 | 1819.47 | 0.33 | 0.744 |
| MAOM | 16.89 | 10.54 | 1.60 | 0.124 |
| pH | −345.05 | 370.02 | −0.93 | 0.362 |
| EC | −3.55 | 3.08 | −1.15 | 0.263 |
